# Supplementary material for: Divergolides T–W with Apoptosis-Inducing Activity from the Mangrove-Derived Actinomycete Streptomyces sp. KFD18
Source: Mar Drugs. 2019 Apr 11;17(4):219. doi: 10.3390/md17040219 (PMC6520978; doi:10.3390/md17040219)
Supplement: Supplementary file 1 [file marinedrugs-17-00219-s001.pdf]

Divergolides T–W with Apoptosis-Inducing Activity from the Mangrove-Derived Actinomycete *Streptomyces* sp. KFD18

Li-Man Zhou [1,2,†], Fan-Dong Kong [2,†], Qing-Yi Xie [2], Qing-Yun Ma [2], Zhong Hu [3] and You-Xing Zhao [2,*] and Du-Qiang Luo [1,*]

1 College of Life Science, Key Laboratory of Medicinal Chemistry and Molecular Diagnosis of Ministry of Education, Hebei University, Baoding 071002, China; zhouliman88@126.com
2 Hainan Key Laboratory for Research and Development of Natural Product from Li Folk Medicine, Institute of Tropical Bioscience and Biotechnology, Chinese Academy of Tropical Agricultural Sciences, Haikou 571101, China; kongfandong@itbb.org.cn (F.-D.K.); xieqingyi@itbb.org.cn (Q.-Y.X.); maqingyun@itbb.org.cn (Q.-Y.M.)
3 Guangdong Provincial Key Laboratory of Marine Biotechnology, Department of Biology, Shantou University, Shantou 515063, China; hzh@stu.edu.cn
* Correspondence: zhaoyouxing@itbb.org.cn (Y.-X.Z.); duqiangluo@163.com (D.-Q.L.); Tel.: +86-139-5169-2350 (Y.-X.Z.)
† These authors contributed equally to this paper

Table of Contents

Figure S1. The 16S rRNA sequence of *streptomyces* sp. KFD18
Figure S2-1. The ^{1}H NMR spectrum of **1**
Figure S2-2. The ^{13}C NMR spectrum of **1**
Figure S2-3. The DEPT spectrum of **1**
Figure S2-4. The HSQC spectrum of **1**
Figure S2-5. The HMBC spectrum of **1**
Figure S2-6. The ^{1}H-^{1}H COSY spectrum of **1**
Figure S2-7. The ROESY spectrum of **1**
Figure S2-8. The IR spectrum of **1**
Figure S2-9. The HRESIMS spectrum of **1**

Figure S3-1. The ^{1}H NMR spectrum of **2**
Figure S3-2. The ^{13}C NMR spectrum of **2**
Figure S3-3. The DEPT spectrum of **2**
Figure S3-4. The HSQC spectrum of **2**
Figure S3-5. The HMBC spectrum of **2**
Figure S3-6. The ^{1}H-^{1}H COSY spectrum of **2**
Figure S3-7. The ROESY spectrum of **2**
Figure S3-8. The IR spectrum of **2**
Figure S3-9. The HRESIMS spectrum of **2**

Figure S4-1. The ^{1}H NMR spectrum of **3**
Figure S4-2. The ^{13}C NMR spectrum of **3**
Figure S4-3. The DEPT spectrum of **3**
Figure S4-4. The HSQC spectrum of **3**
Figure S4-5. The HMBC spectrum of **3**
Figure S4-6. The ^{1}H-^{1}H COSY spectrum of **3**
Figure S4-7. The ROESY spectrum of **3**
Figure S4-8. The IR spectrum of **3**
Figure S4-9. The HRESIMS spectrum of **3**

Figure S5-1. The ^{1}H NMR spectrum of **4**
Figure S5-2. The ^{13}C NMR spectrum of **4**
Figure S5-3. The DEPT spectrum of **4**
Figure S5-4. The HSQC spectrum of **4**
Figure S5-5. The HMBC spectrum of **4**
Figure S5-6. The ^{1}H-^{1}H COSY spectrum of **4**
Figure S5-7. The ROESY spectrum of **4**
Figure S5-8. The IR spectrum of **4**
Figure S5-9. The HRESIMS spectrum of **4**

TTACCTGCAAGTCGAACGATGAACCACTTCGGTGGGGATTAGTGGCGAACGGGTGAGTAACACGTGG

GCAATCTGCCCTGCACTCTGGGACAAGCCCTGGAAACGGGGTCTAATACCGGATATTGACCTTCACGG

GCATCTGTGAGGTTCGAAAGCTCCGGCGGTGCAGGATGAGCCCGCGGCCTATCAGCTTGTTGGTGAGG

TAATGGCTCACCAAGGCGACGACGGGTAGCCGGCCTGAGAGGGCGACCGGCCACACTGGGACTGAG

ACACGGCCCAGACTCCTACGGGAGGCAGCAGTGGGGAATATTGCACAATGGGCGAAAGCCTGATGCA

GCGACGCCGCGTGAGGGATGACGGCCTTCGGGTTGTAAACCTCTTTCAGCAGGGAAGAAGCGAAAGT

GACGGTACCTGCAGAAGAAGCGCCGGCTAACTACGTGCCAGCAGCCGCGGTAATACGTAGGGCGCAA

GCGTTGTCCGGAATTATTGGGCGTAAAGAGCTCGTAGGCGGCTTGTCACGTCGGTTGTGAAAGCCCGG

GGCTTAACCCCGGGTCTGCAGTCGATACGGGCAGGCTAGAGTTCGGTAGGGGAGATCGGAATTCCTGG

TGTAGCGGTGAAATGCGCAGATATCAGGAGGAACACCGGTGGCGAAGGCGGATCTCTGGGCCGATAC

TGACGCTGAGGAGCGAAAGCGTGGGGAGCGAACAGGATTAGATACCCTGGTAGTCCACGCCGTAAAC

GGTGGGCACTAGGTGTGGGCAACATTCCACGTTGTCCGTGCCGCAGCTAACGCATTAAGTGCCCCGCC

TGGGGAGTACGGCCGCAAGGCTAAAACTCAAAGGAATTGACGGGGGCCCGCACAAGCGGCGGAGCA

TGTGGCTTAATTCGACGCAACGCGAAGAACCTTACCAAGGCTTGACATACACCGGAAACGGCCAGAG

ATGGTCGCCCCCTTGTGGTCGGTGTACAGGTGGTGCATGGCTGTCGTCAGCTCGTGTCGTGAGATGTT

GGGTTAAGTCCCGCAACGAGCGCAACCCTTGTCCCGTGTTGCCAGCAAGCTCCTTCGGGGGTGTTGG

GGACTCACGGGAGACCGCCGGGGTCAACTCGGAGGAAGGTGGGGACGACGTCAAGTCATCATGCCC

CTTATGTCTTGGGCTGCACACGTGCTACAATGGCCGGTACAATGAGCTGCGATACCGCAAGGTGGAGC

GAATCTCAAAAAGCCGGTCTCAGTTCGGATTGGGGTCTGCAACTCGACCCCATGAAGTCGGAGTCGCT

AGTAATCGCAGATCAGCATTGCTGCGGTGAATACGTTCCCGGGCCTTGTACACACCGCCCGTCACGTC

ACGAAAGTCGGTAACACCCGAAGCCGGTGGCCCAACCCC

Figure S1. The 16S rRNA sequence of *streptomyces* sp. KFD18

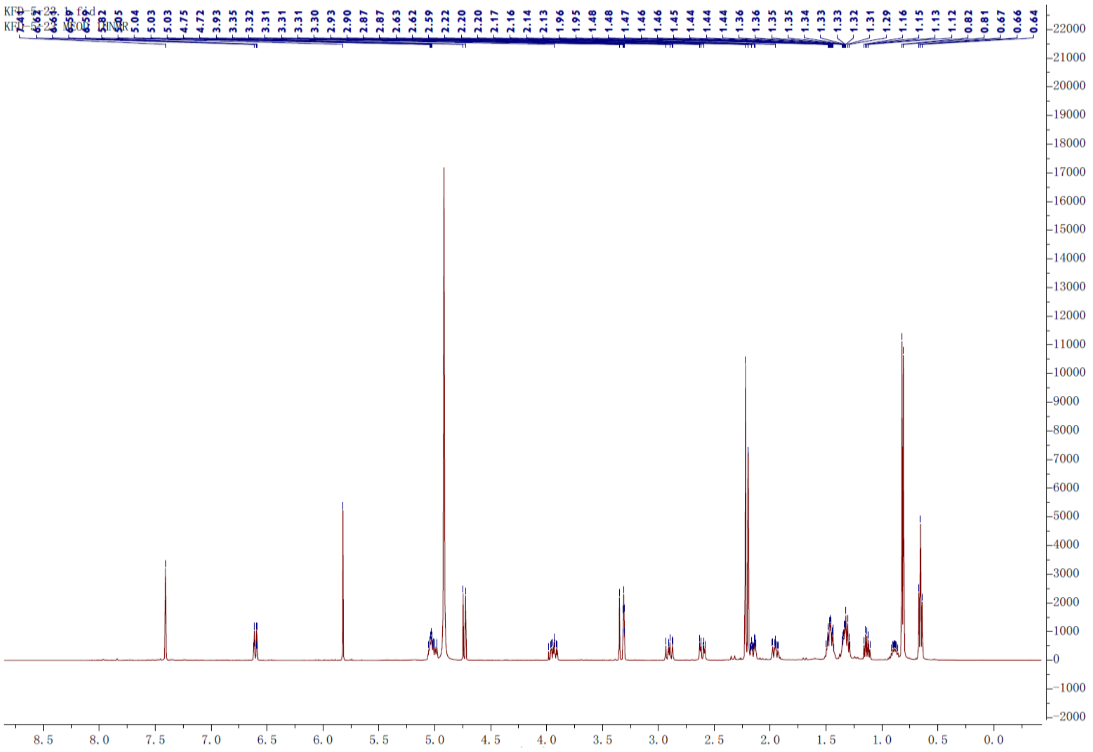

Figure S2-1. The ^{1}H NMR spectrum of **1**

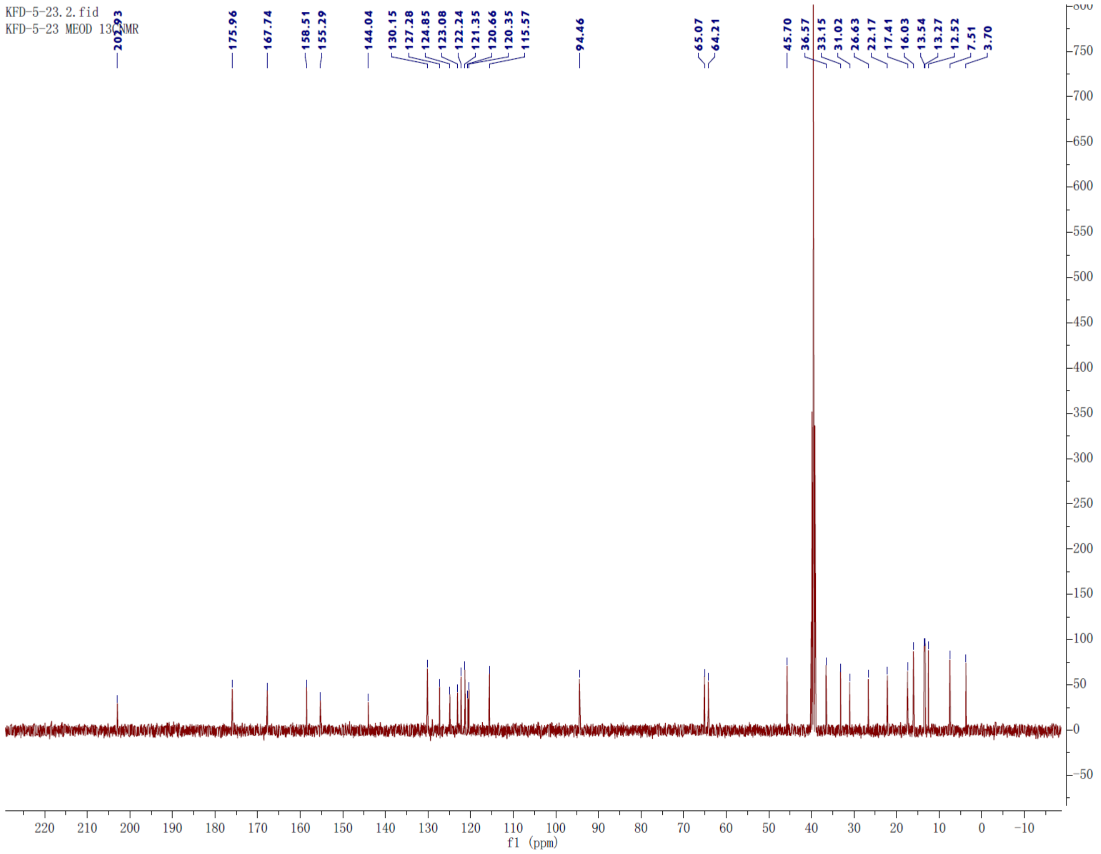

Figure S2-2. The ^{13}C NMR spectrum of **1**

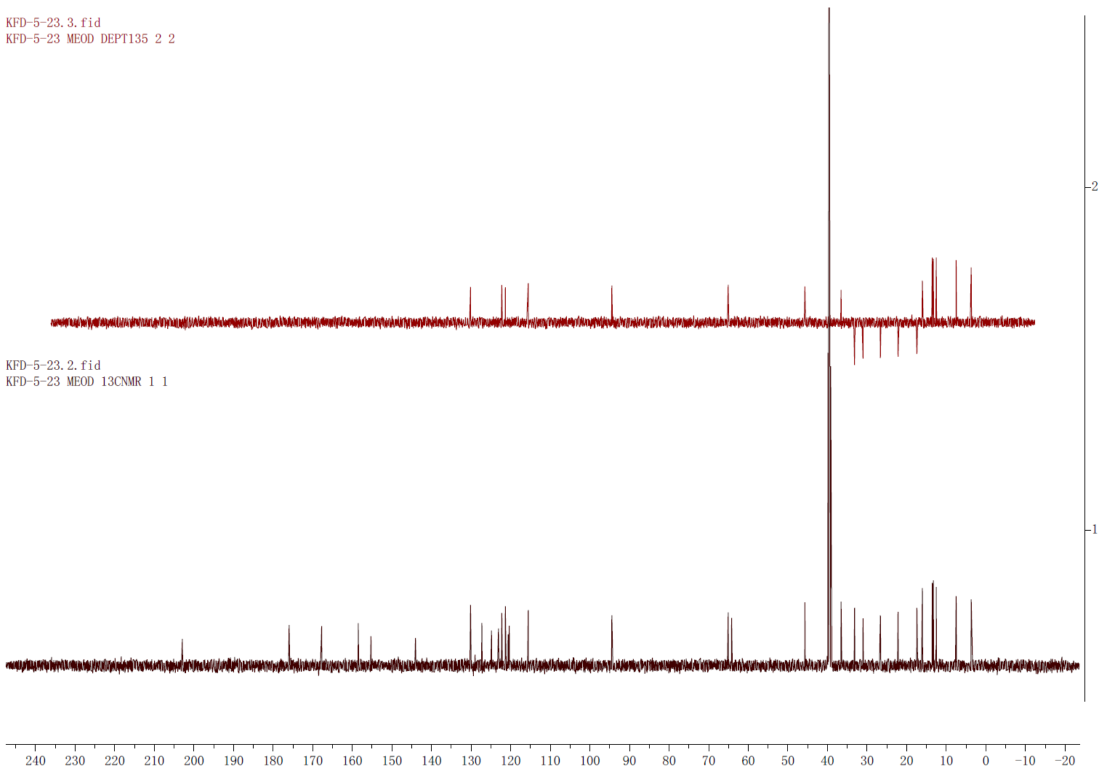

Figure S2-3. The DEPT spectrum of **1**

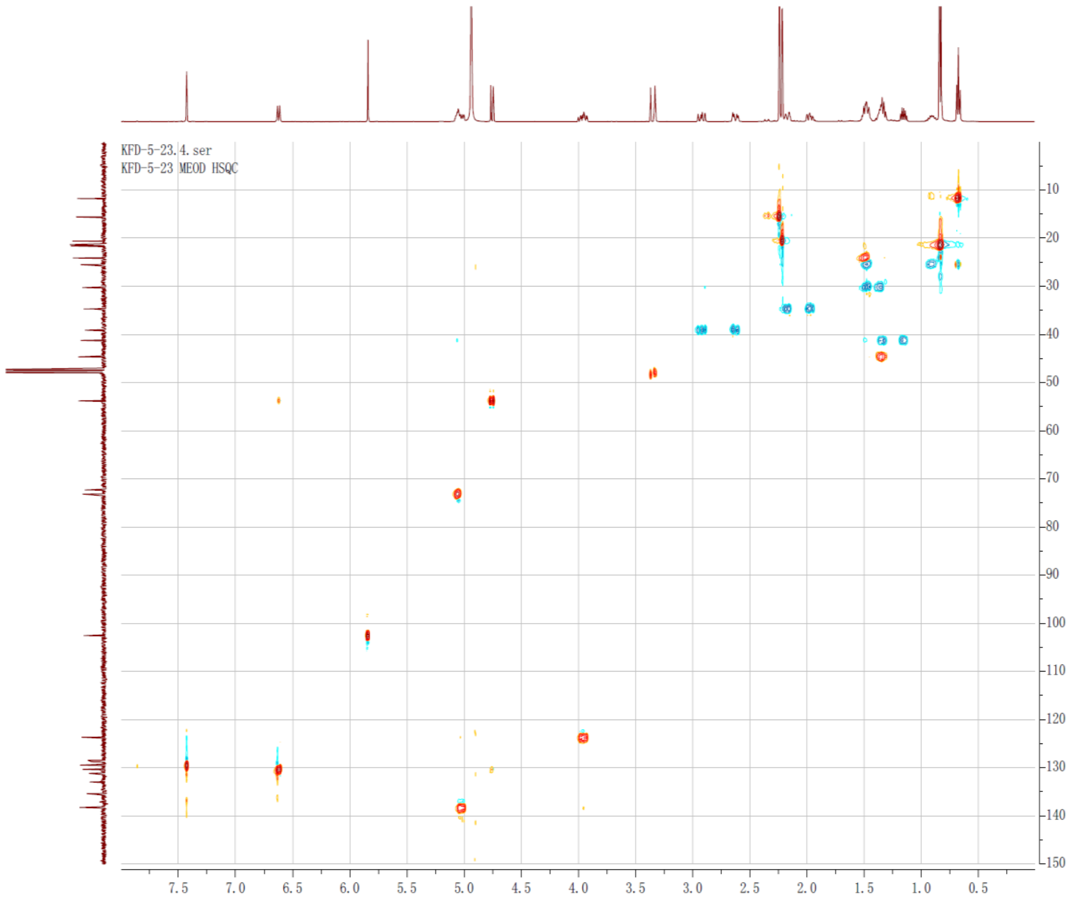

Figure S2-4. The HSQC spectrum of **1**

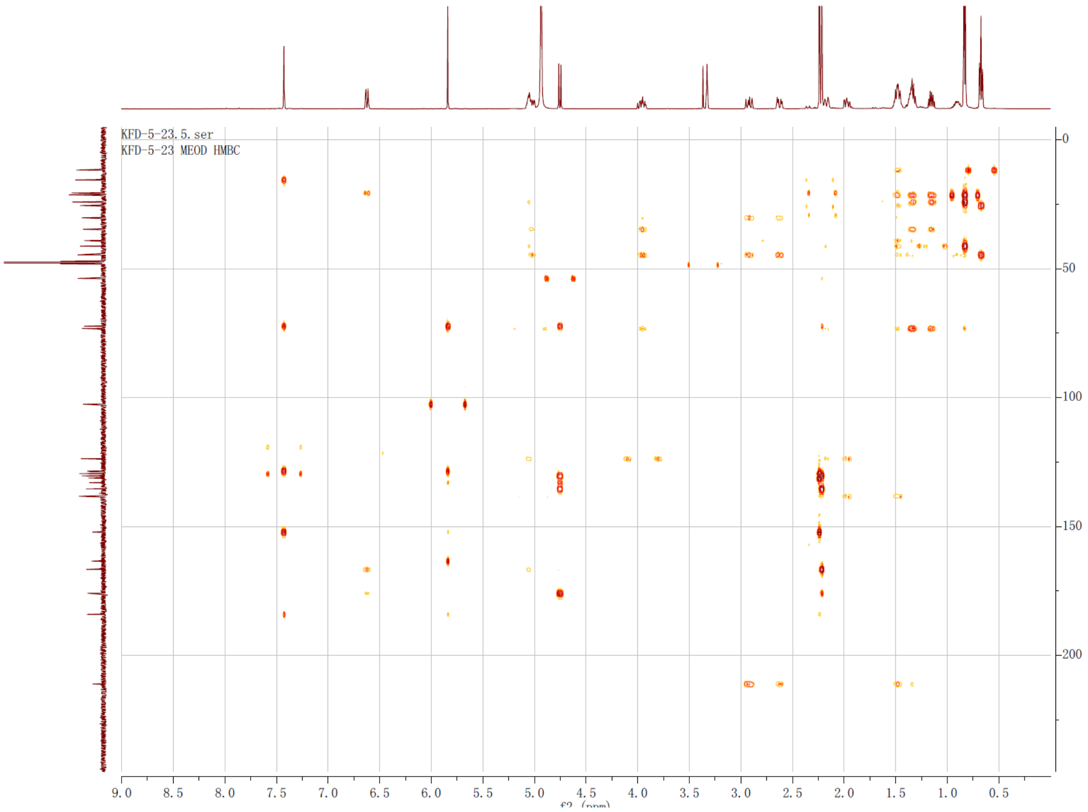

Figure S3-5. The HMBC spectrum of **2**

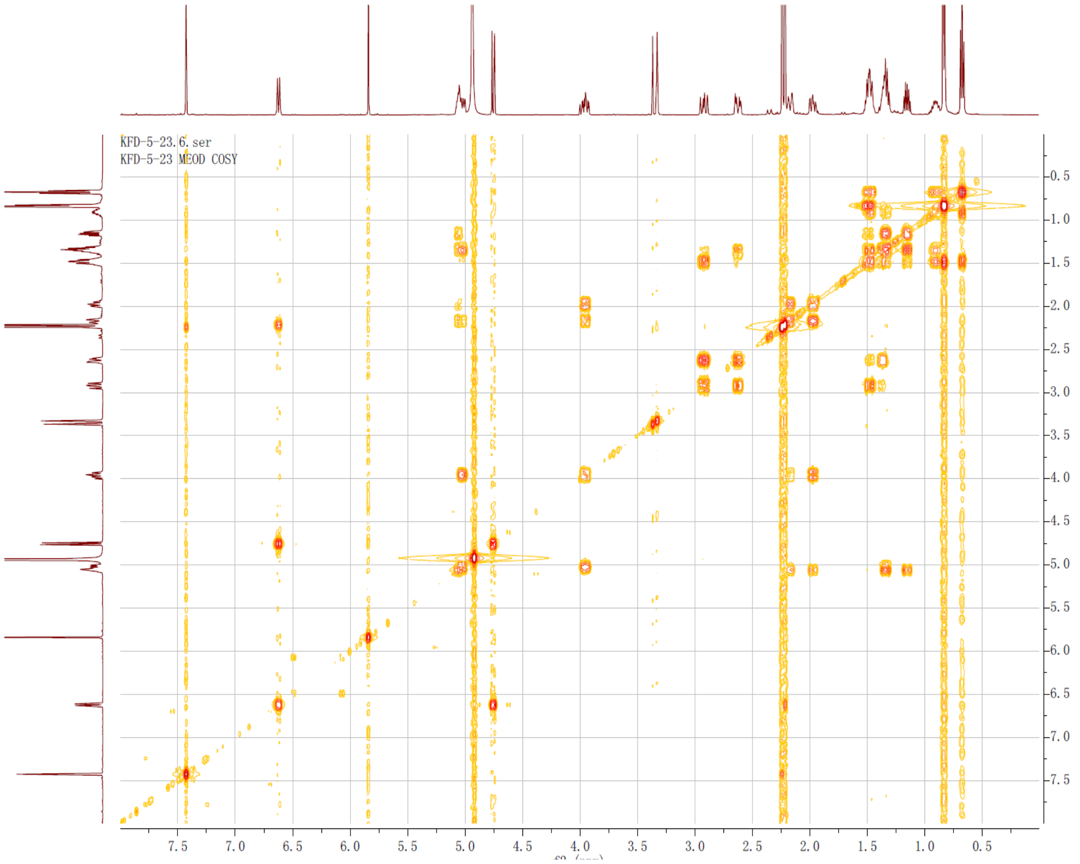

Figure S2-6. The ^{1}H-^{1}H COSY spectrum of **1**

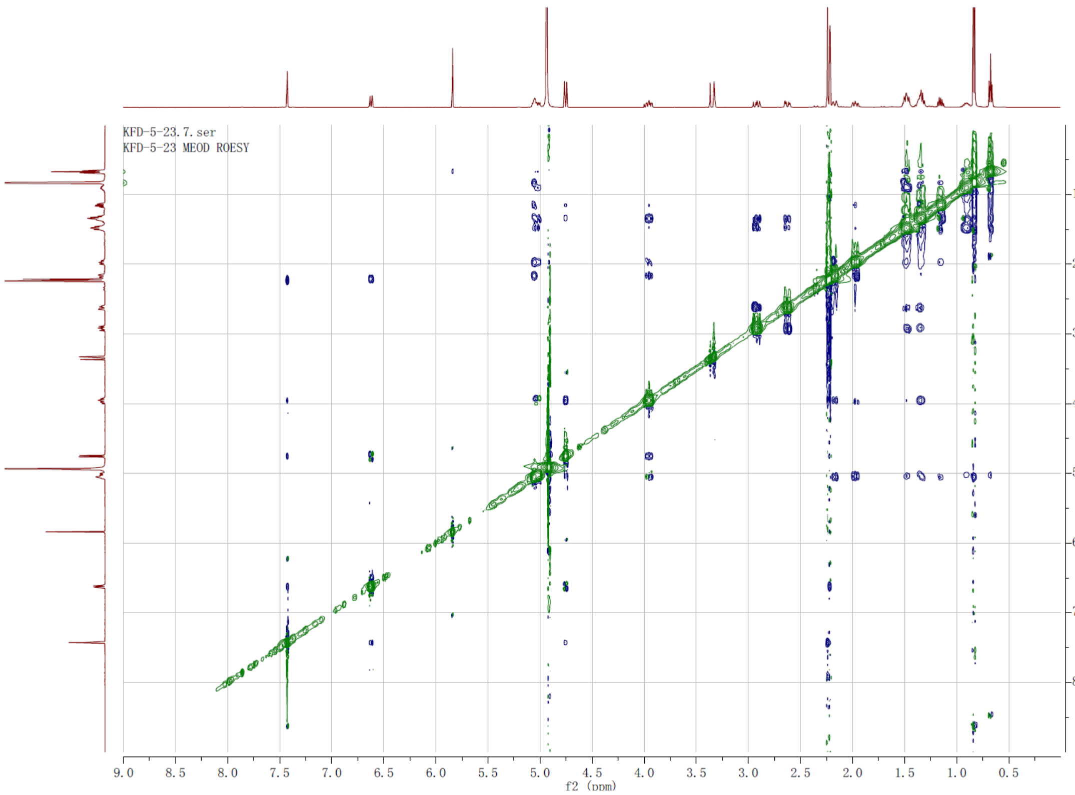

Figure S2-7. The ROESY spectrum of **1**

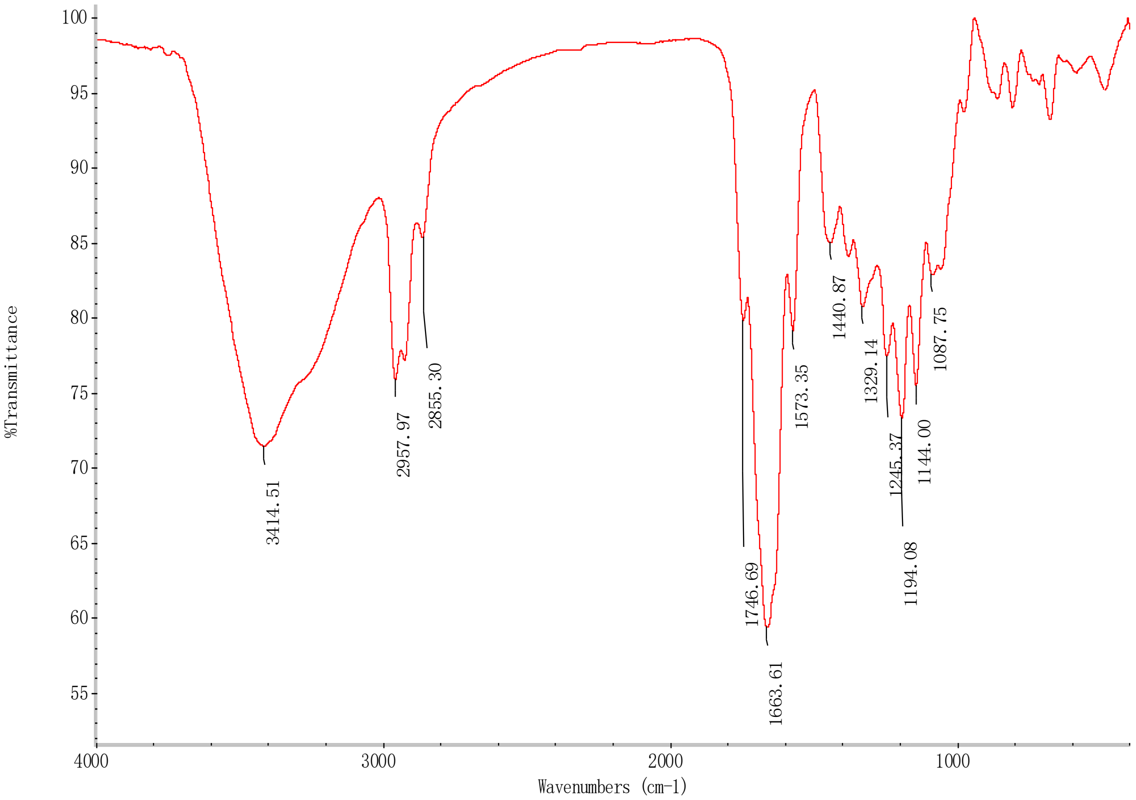

Figure S2-9. The IR spectrum of **1**

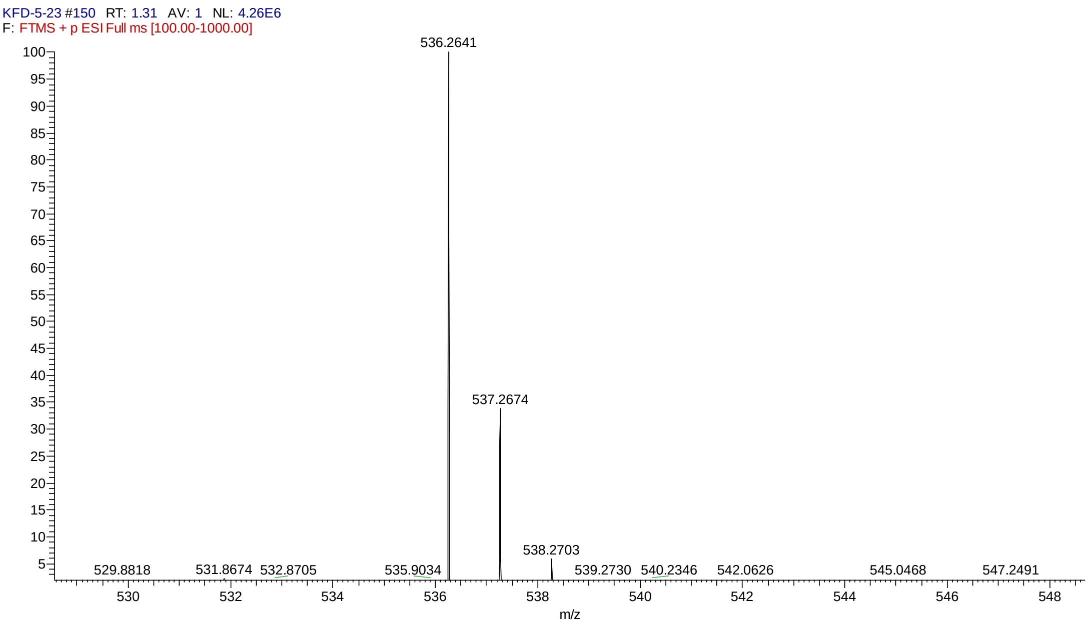

Idx	Formula	RDB	Delta ppm
1	$C_{31}H_{38}O_7N$	13.5	-0.334

Caculated 536.2643 Found 536.2641

Figure S2-9. The HRESIMS spectrum of **1**

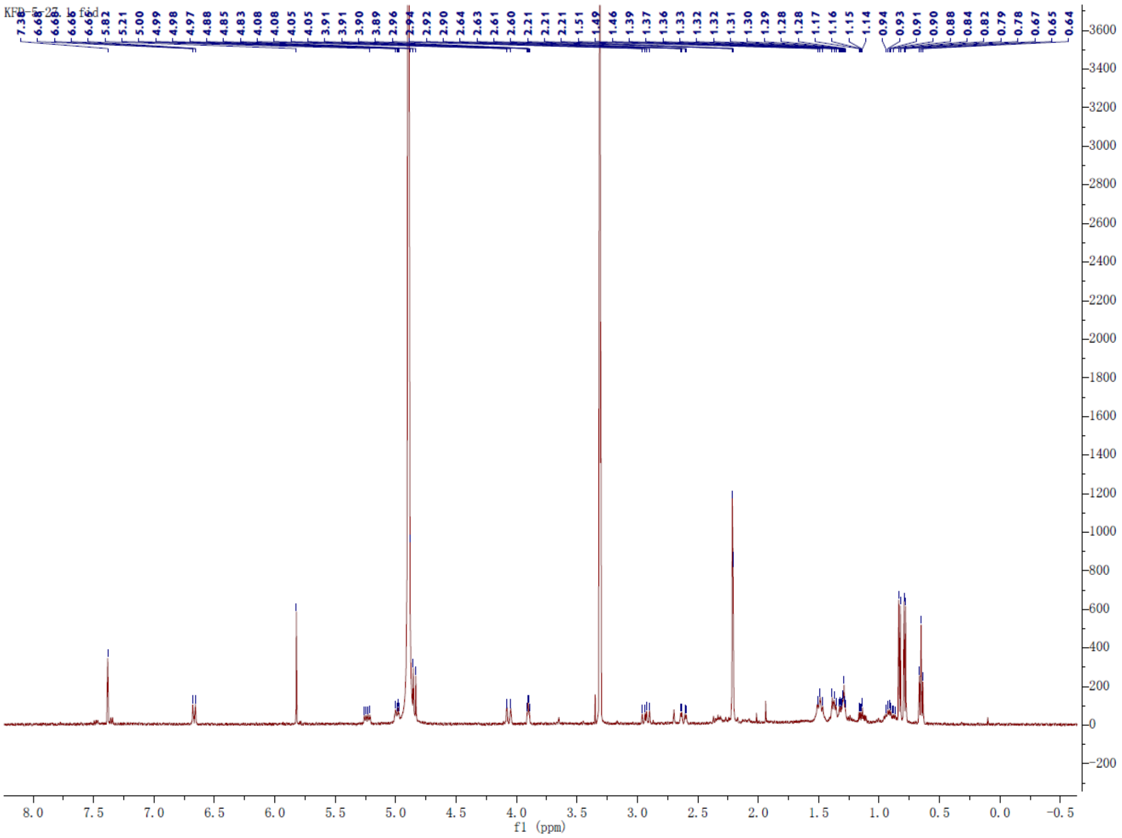

Figure S3-1. The ^{1}H NMR spectrum of **2**

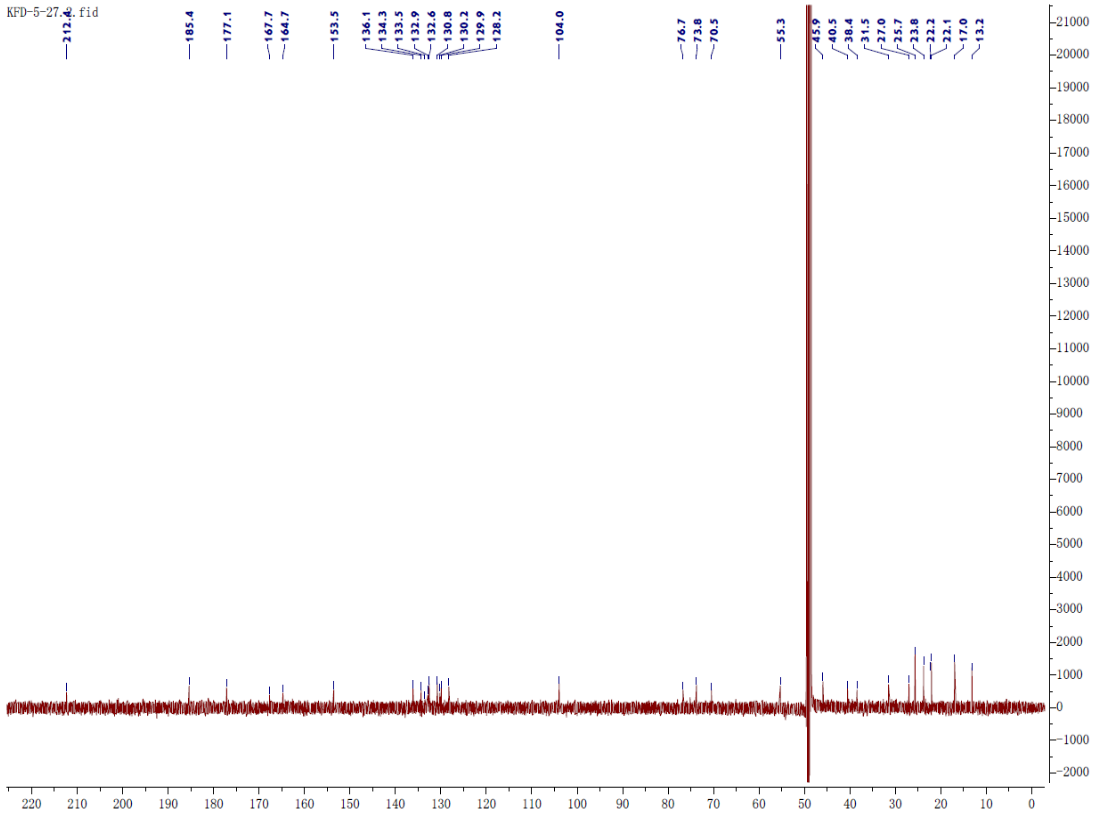

Figure S3-2. The ^{13}C NMR spectrum of **2**

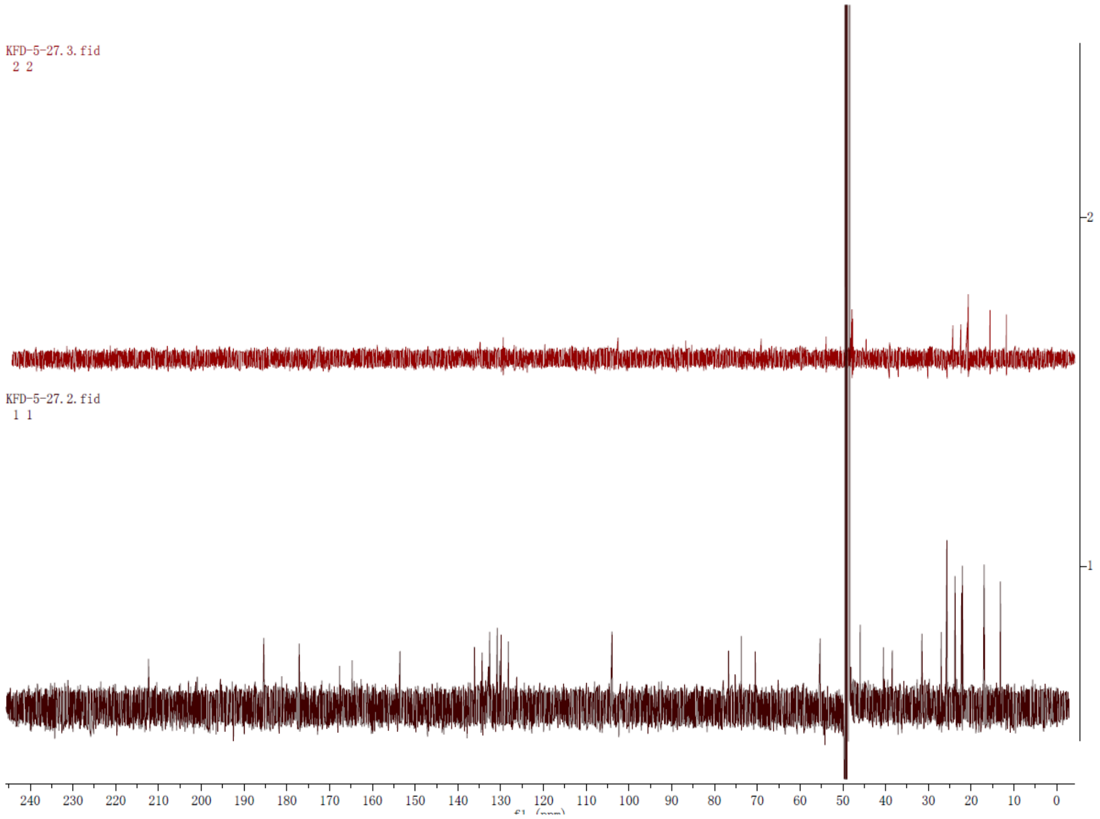

Figure S3-3. The DEPT spectrum of **2**

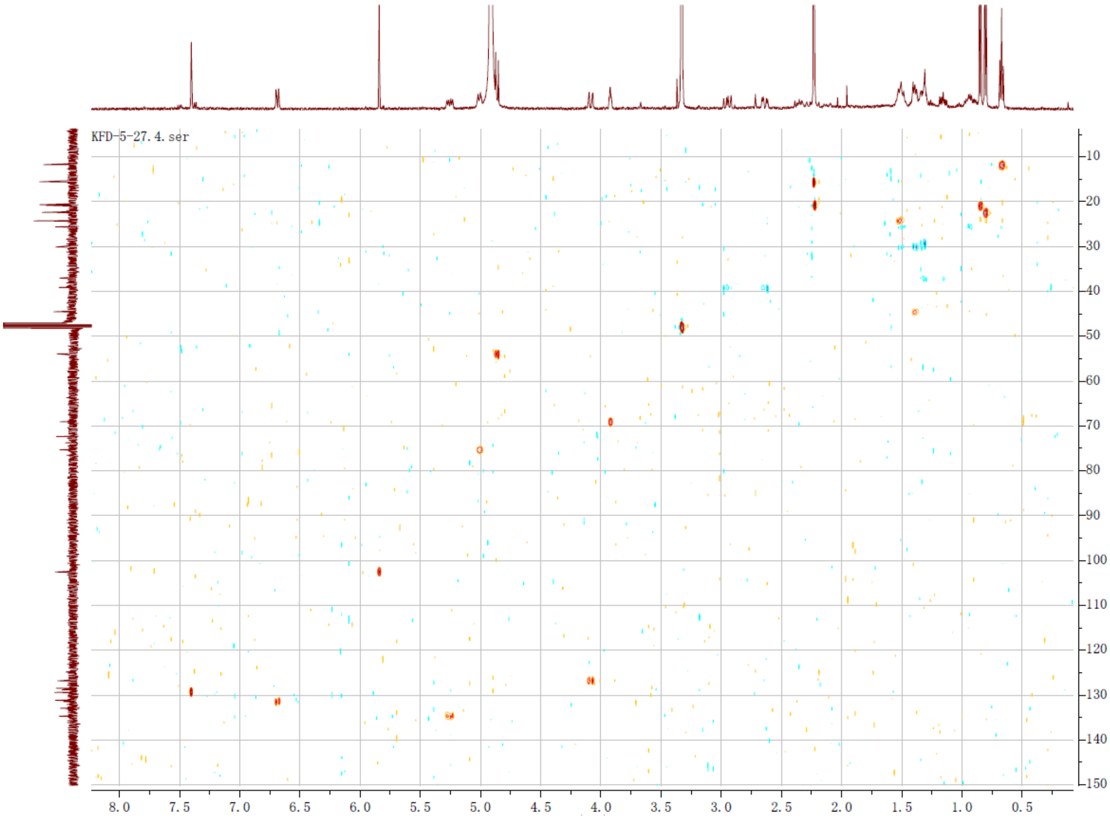

Figure S3-4. The HSQC spectrum of **2**

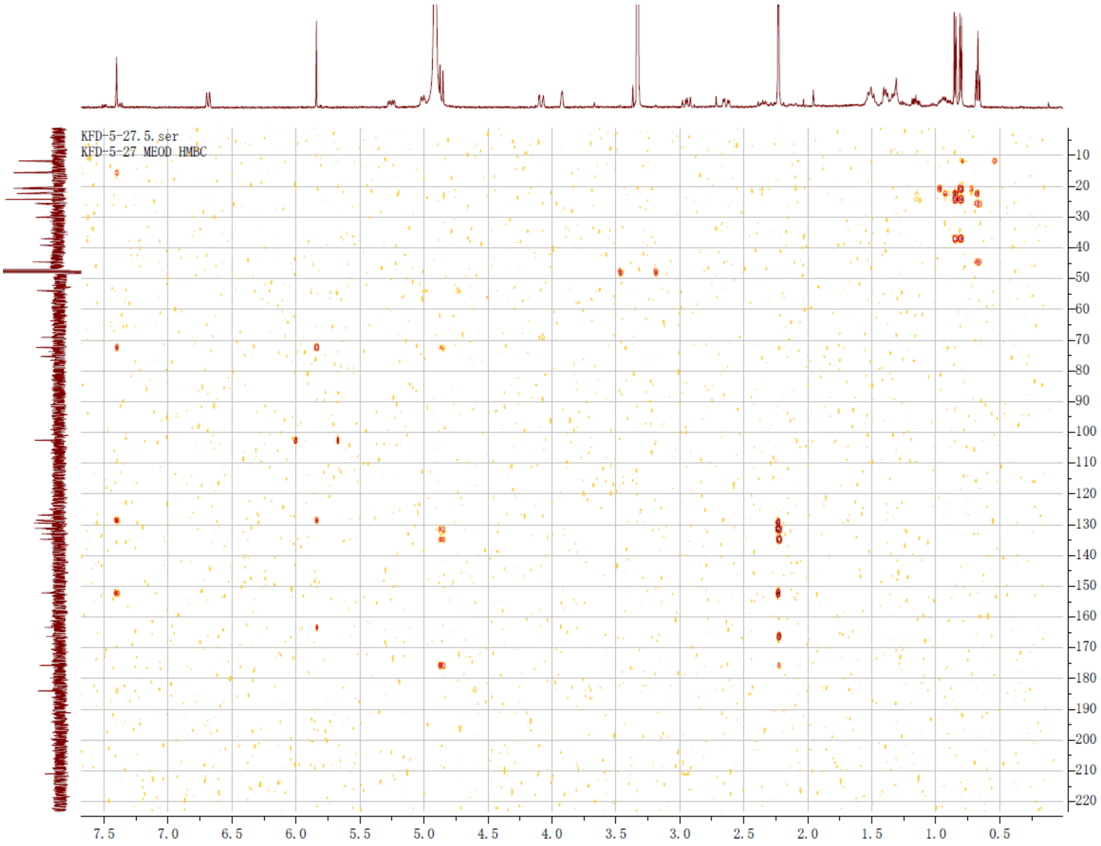

Figure S3-5. The HMBC spectrum of **2**

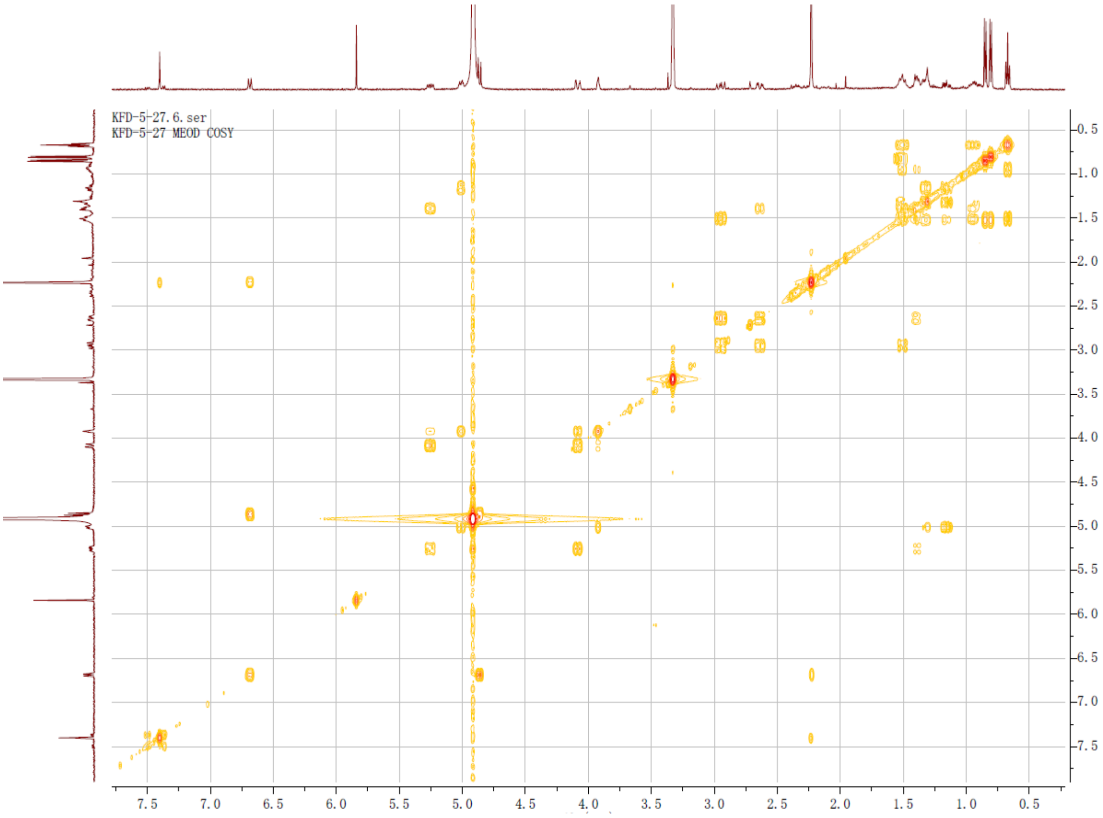

Figure S3-6. The ^{1}H-^{1}H COSY spectrum of **2**

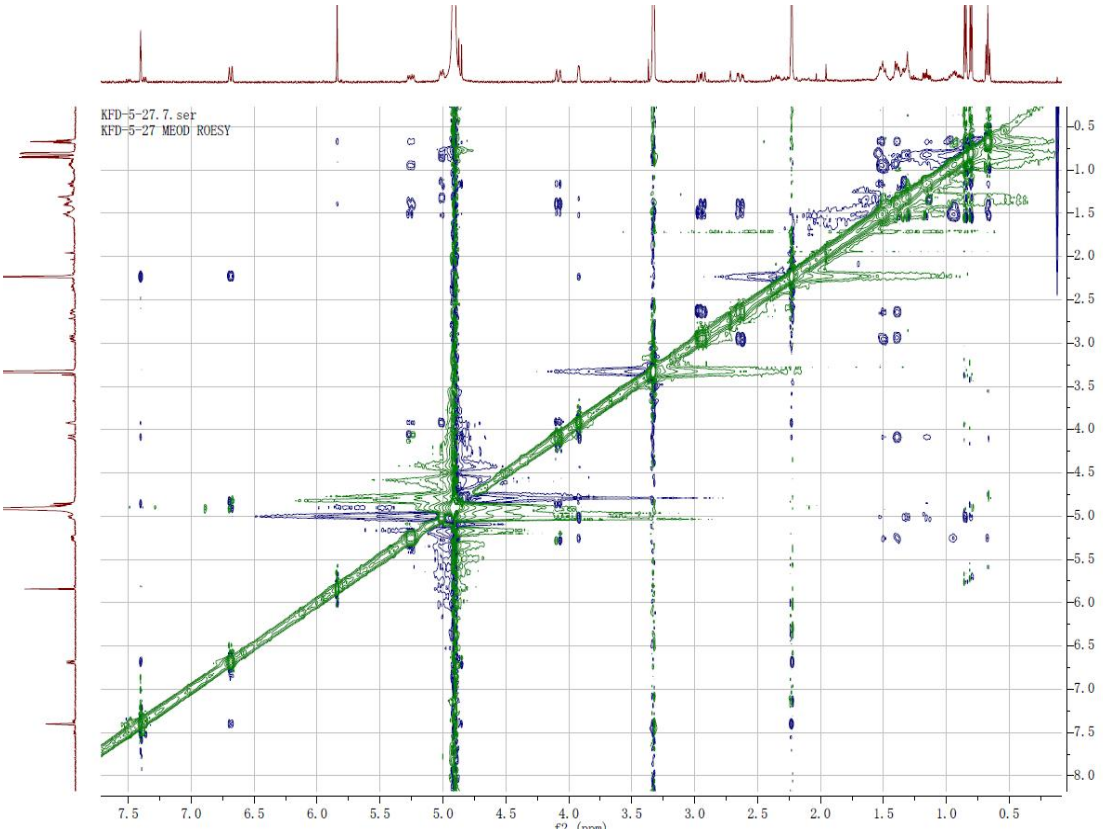

Figure S3-7. The ROESY spectrum of **2**

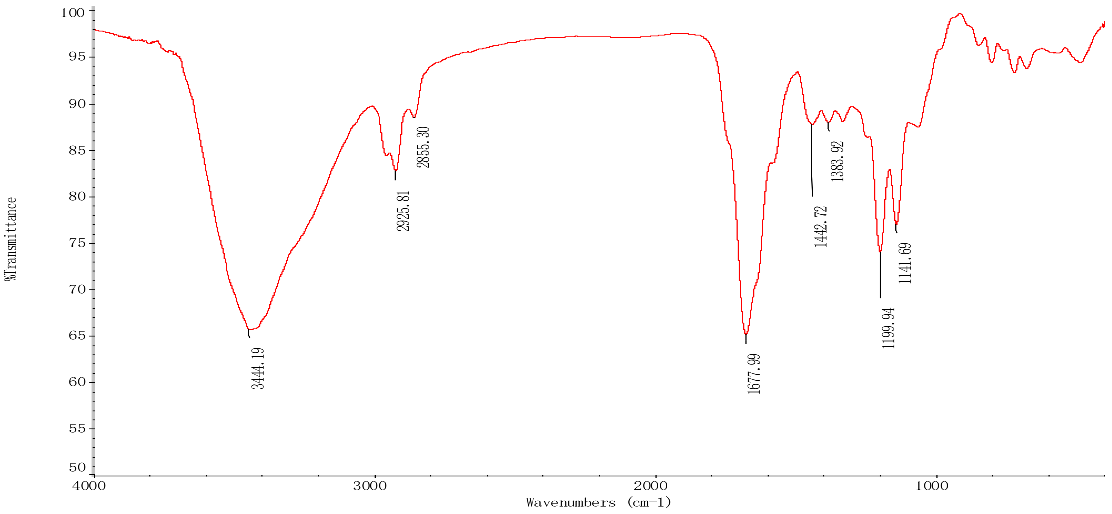

Figure S3-8. The IR spectrum of **2**

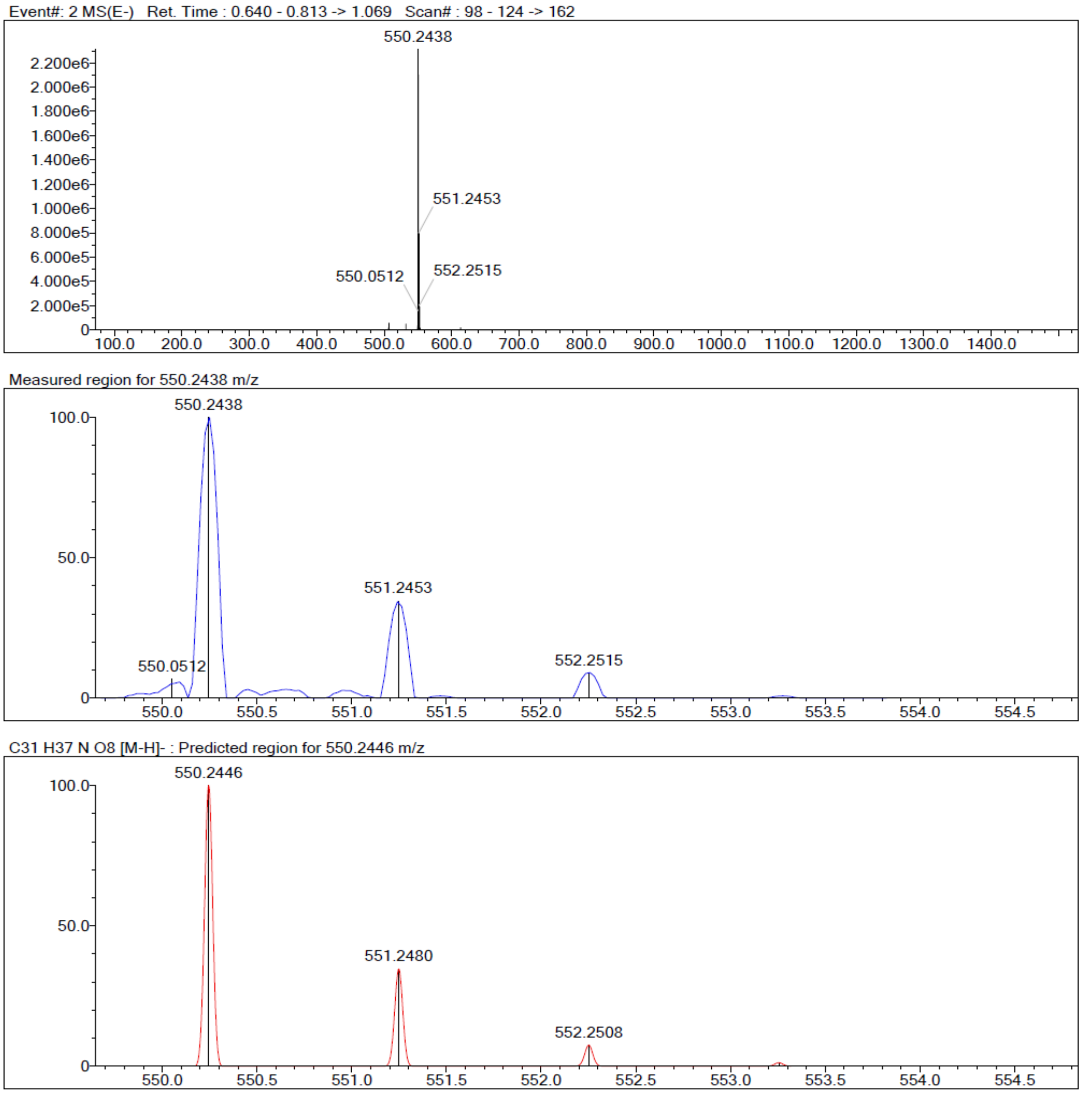

Rank	Score	Formula (M)	Ion	Meas. m/z	Pred. m/z	Df. (mDa)	Df. (ppm)	Iso	DBE
1	80.20	C31 H37 N O8	[M-H]-	550.2438	550.2446	-0.8	-1.45	81.11	14.0

Figure S3-9. The HRESIMS spectrum of **2**

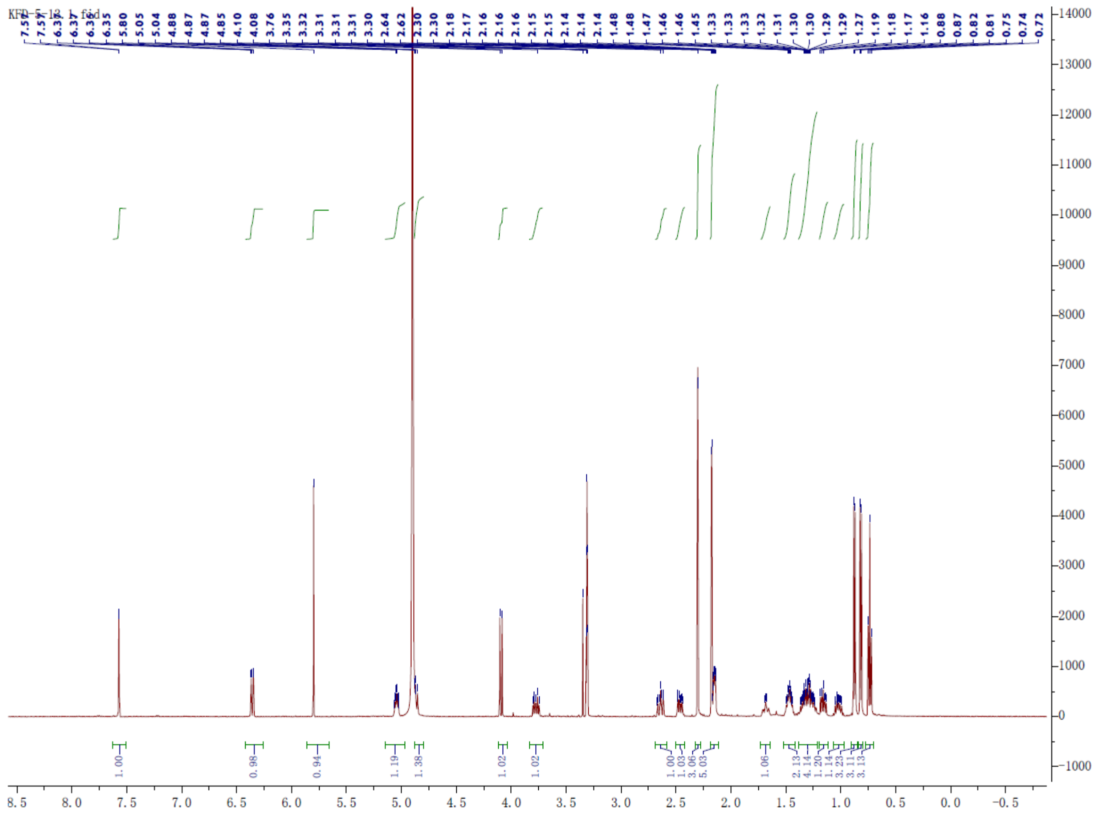

Figure S4-1. The ^{1}H NMR spectrum of **3**

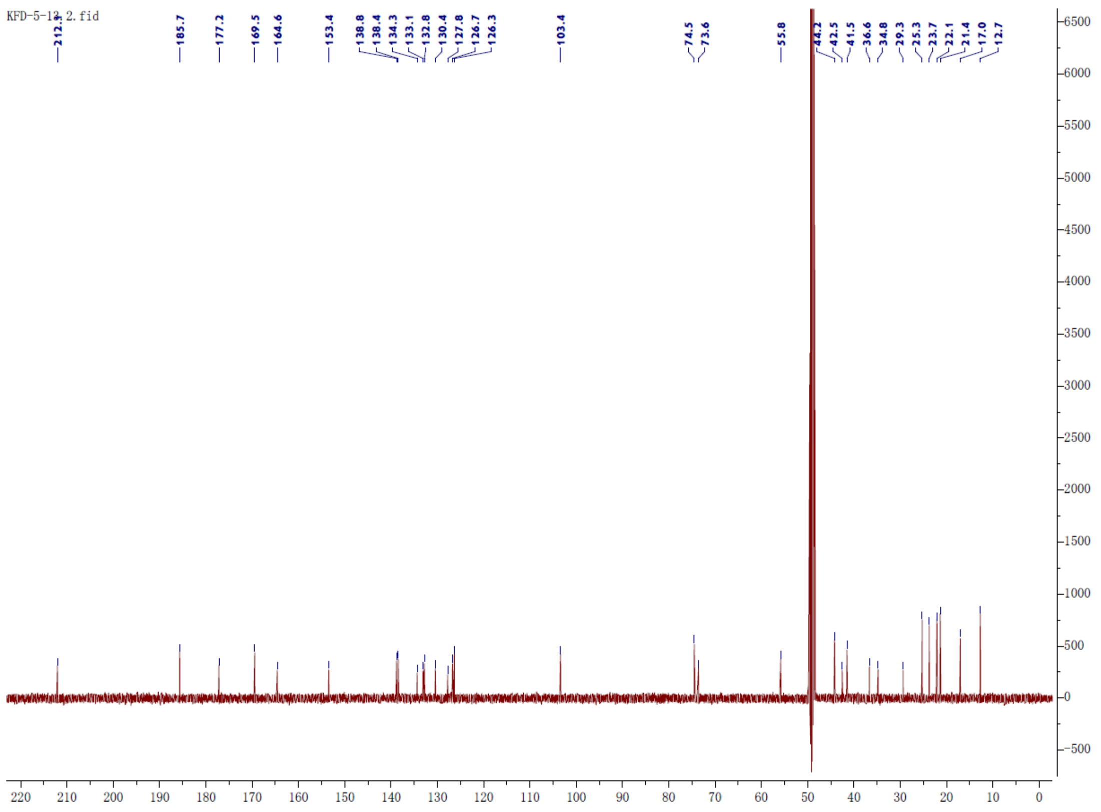

Figure S4-2. The ^{13}C NMR spectrum of **3**

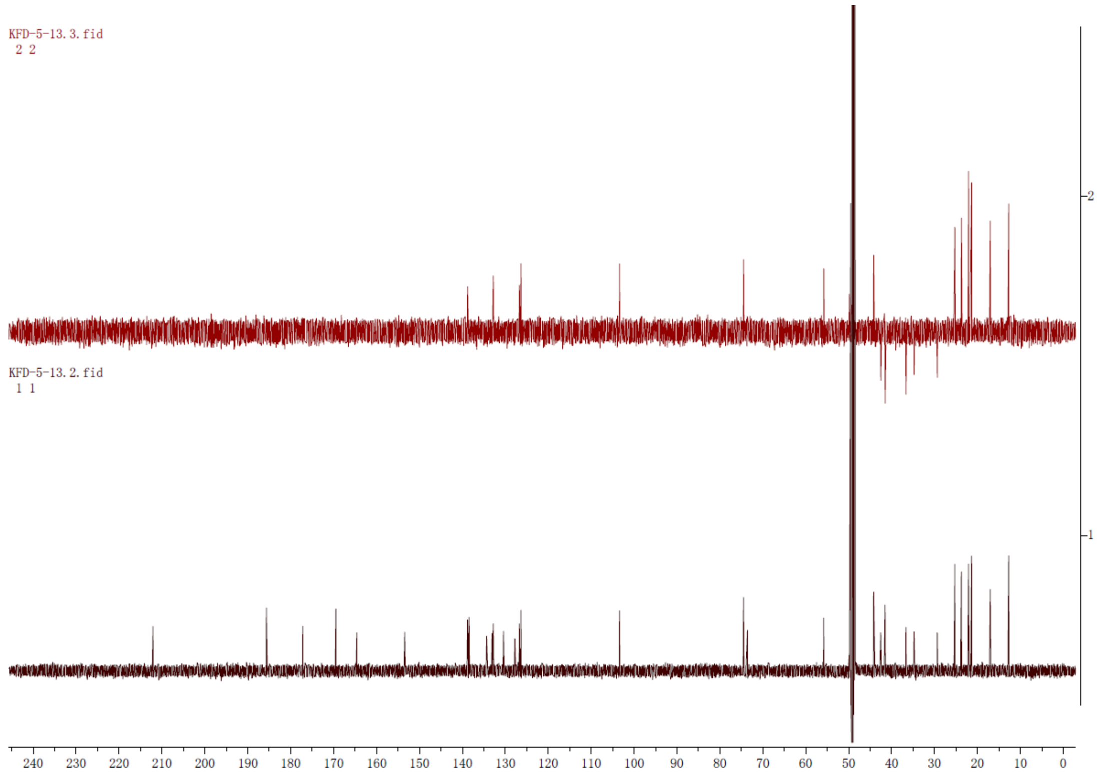

Figure S4-3. The DEPT spectrum of **3**

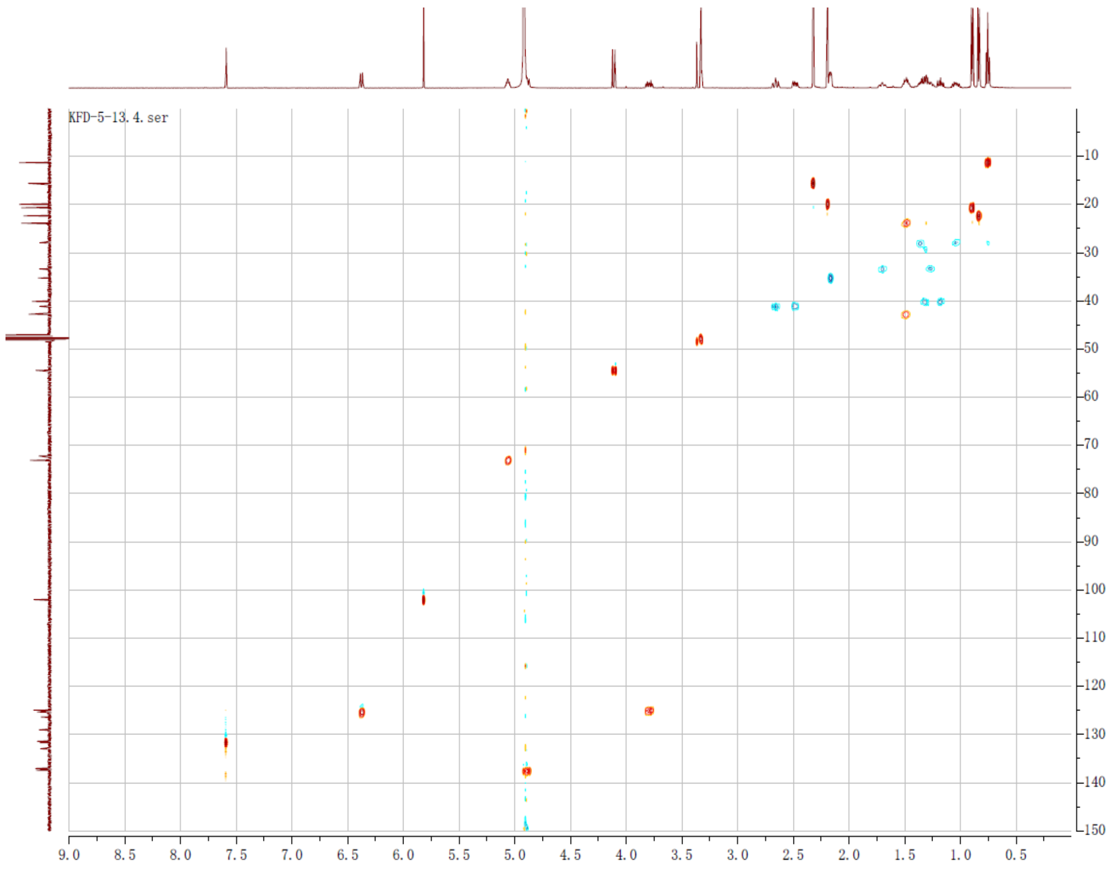

Figure S4-4. The HSQC spectrum of **3**

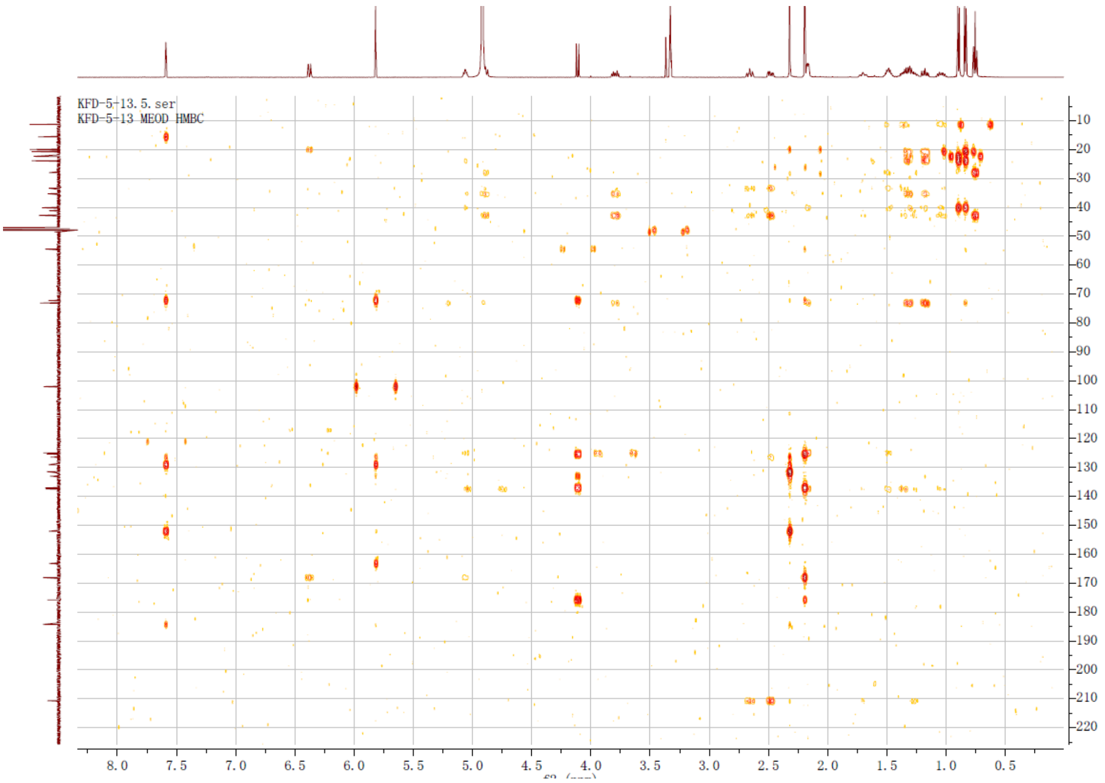

Figure S4-5. The HMBC spectrum of **3**

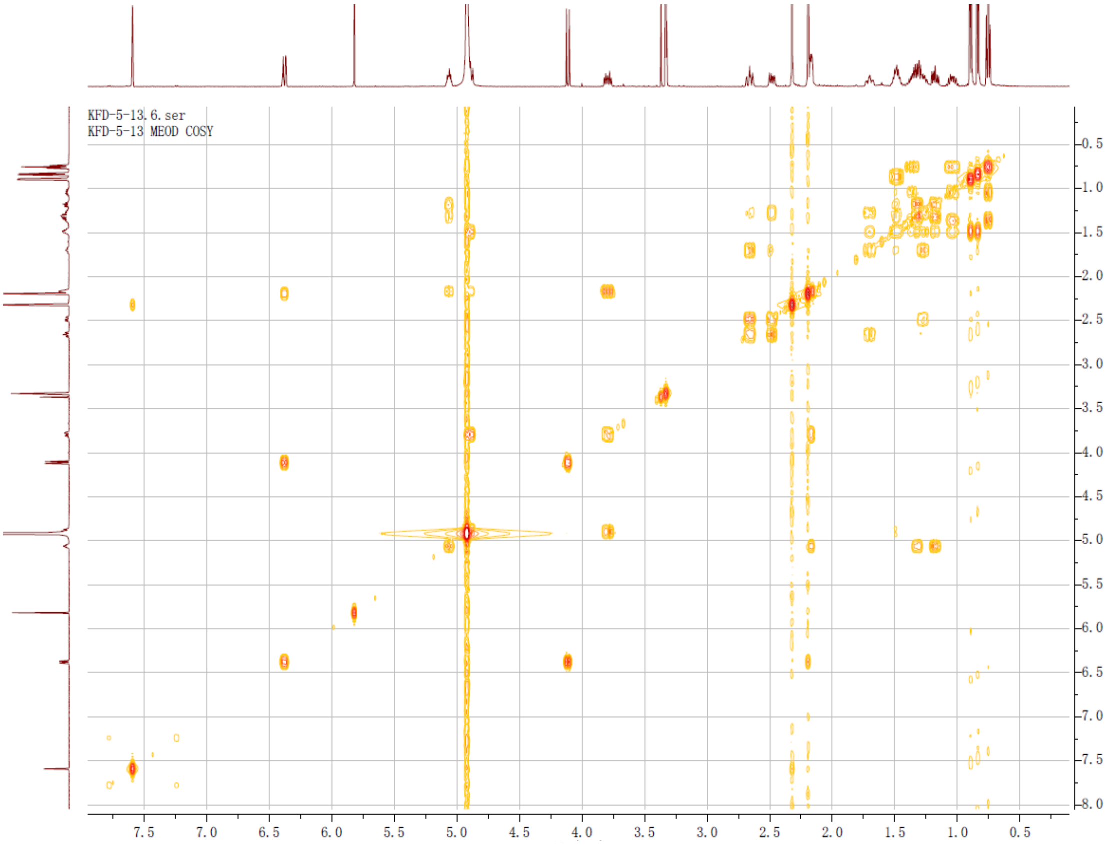

Figure S4-6. The ^{1}H-^{1}H COSY spectrum of **3**

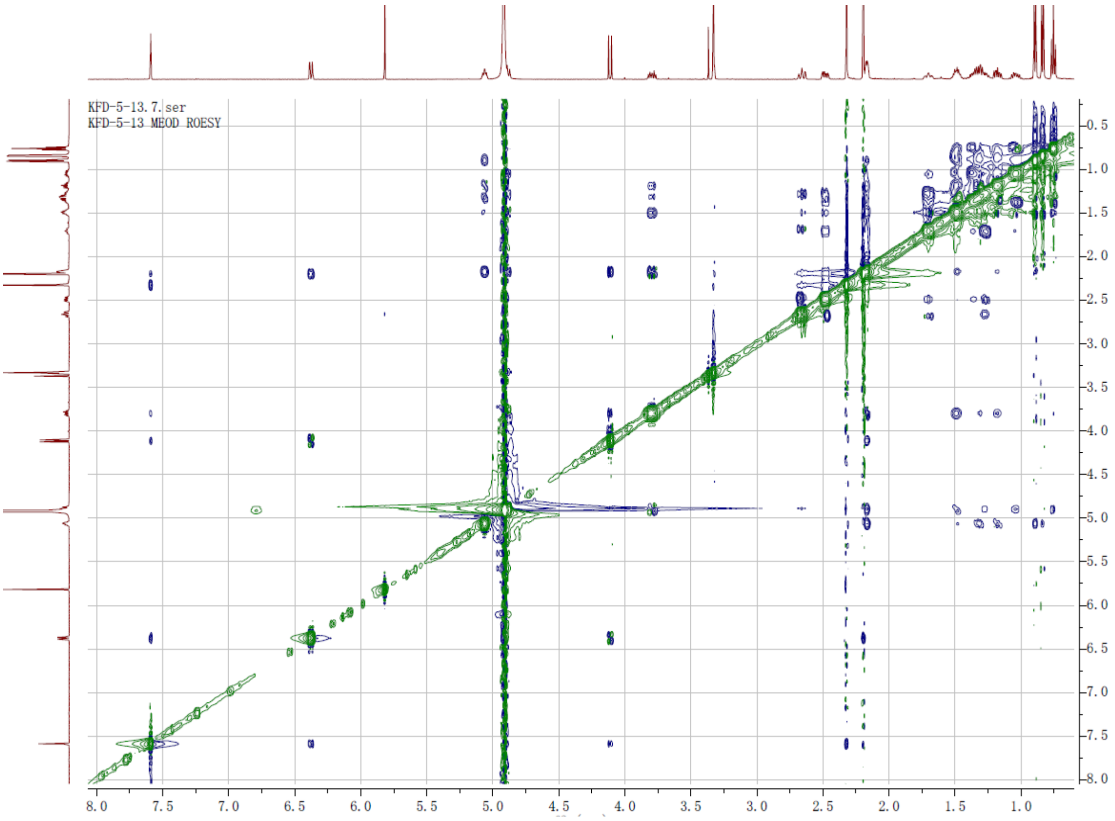

Figure S4-7. The ROESY spectrum of **3**

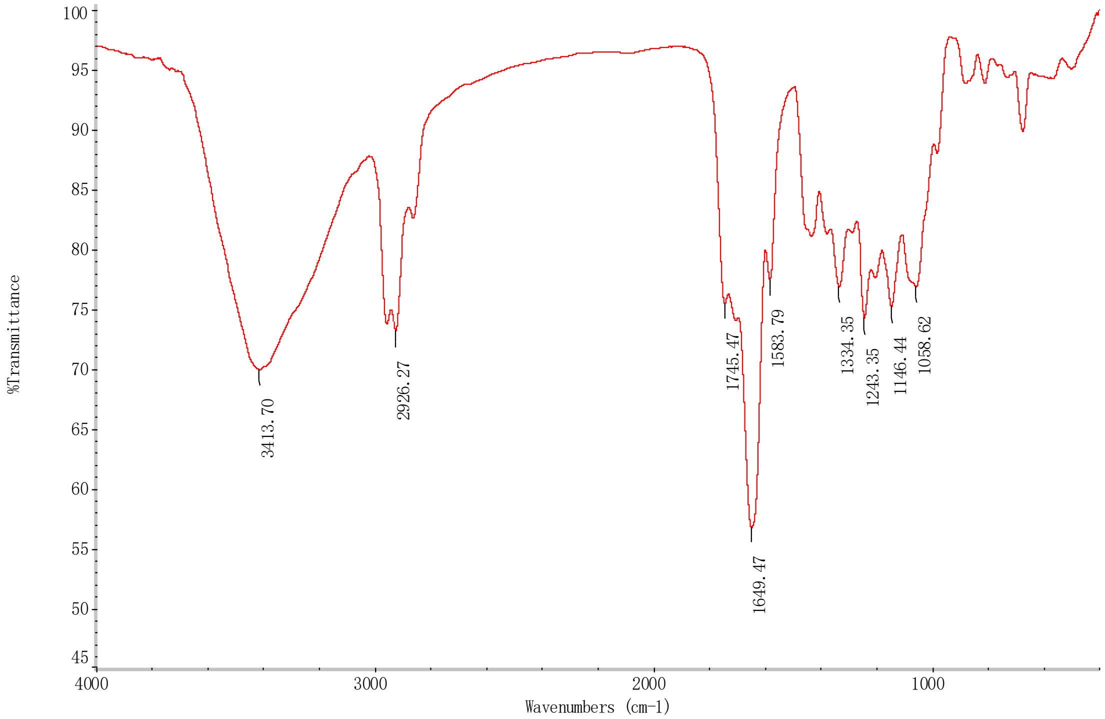

Figure S4-8. The IR spectrum of **3**

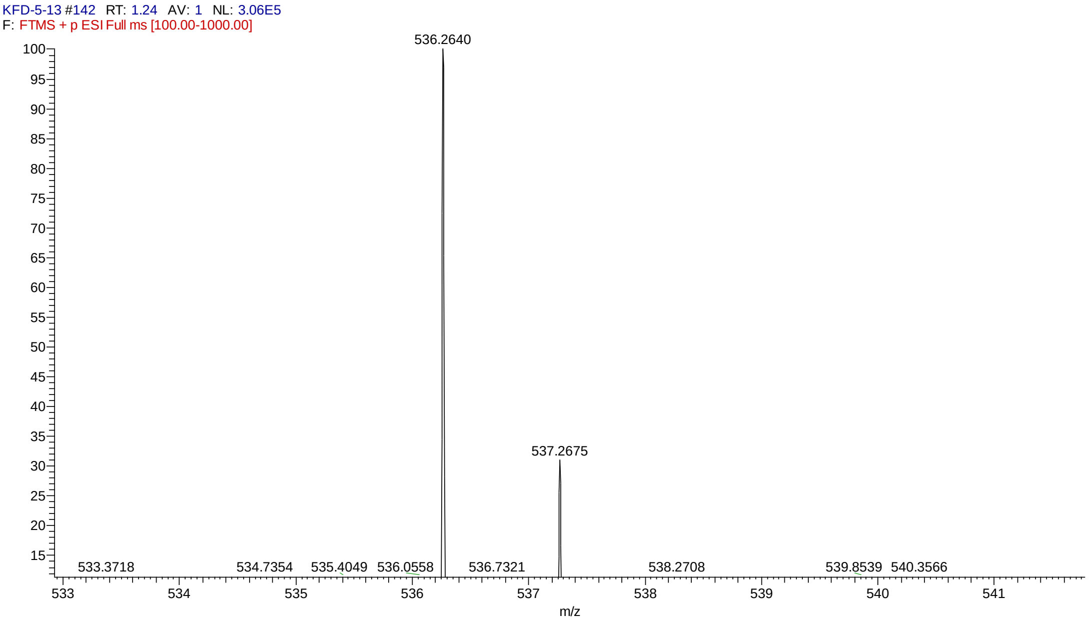

Idx	Formula	RDB	Delta ppm
1	$C_{31}H_{38}O_7N$	13.5	-0.446

Caculated 536.2643 Found 536.2640

Figure S4-9. The HRESIMS spectrum of **3**

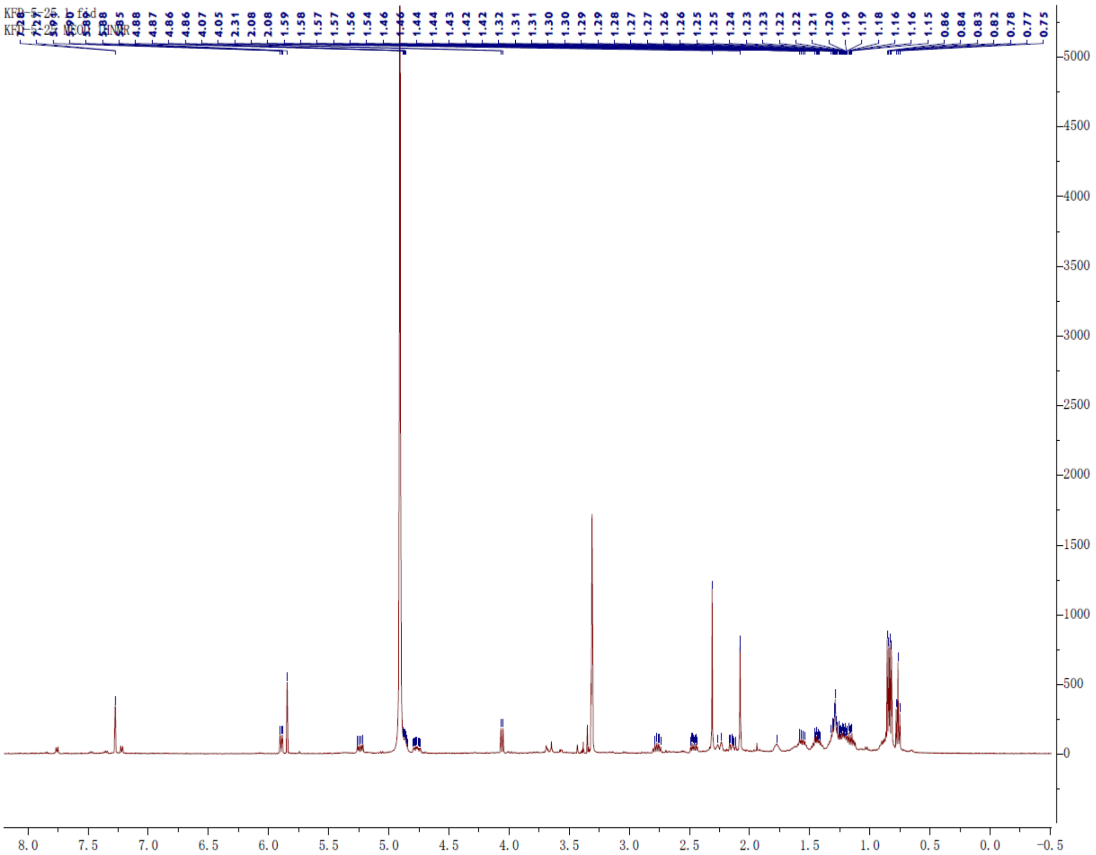

Figure S5-1. The ^{1}H NMR spectrum of **4**

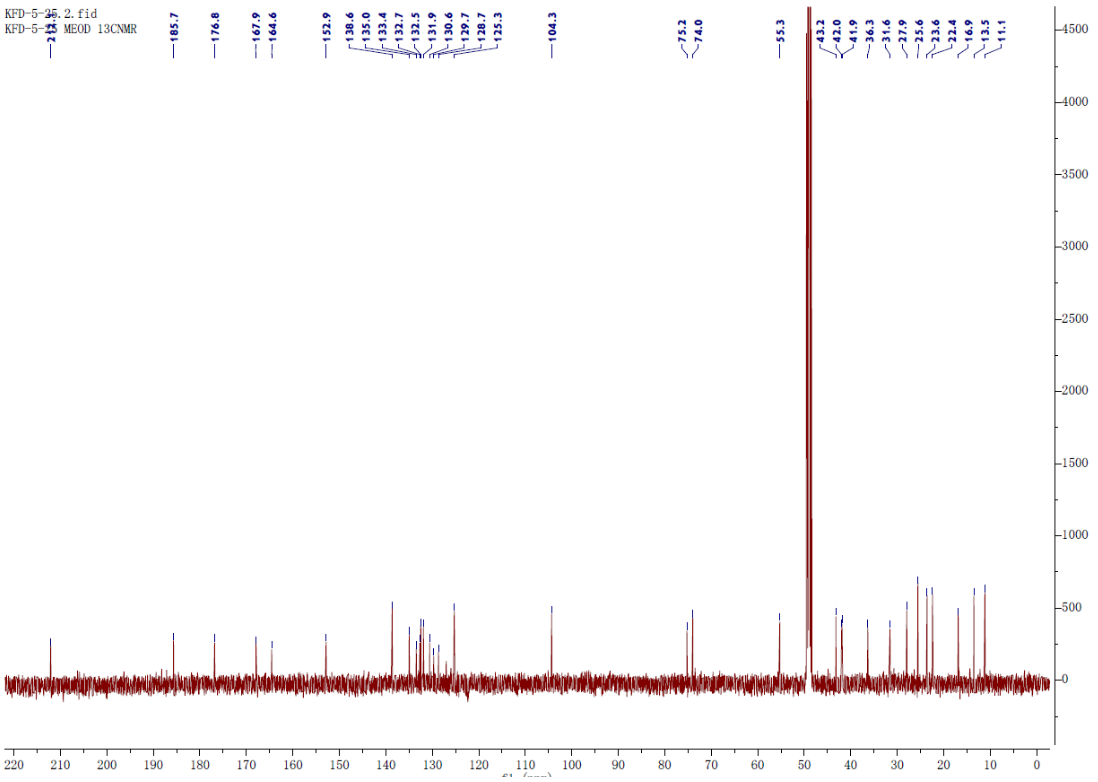

Figure S5-2. The ^{13}C NMR spectrum of **4**

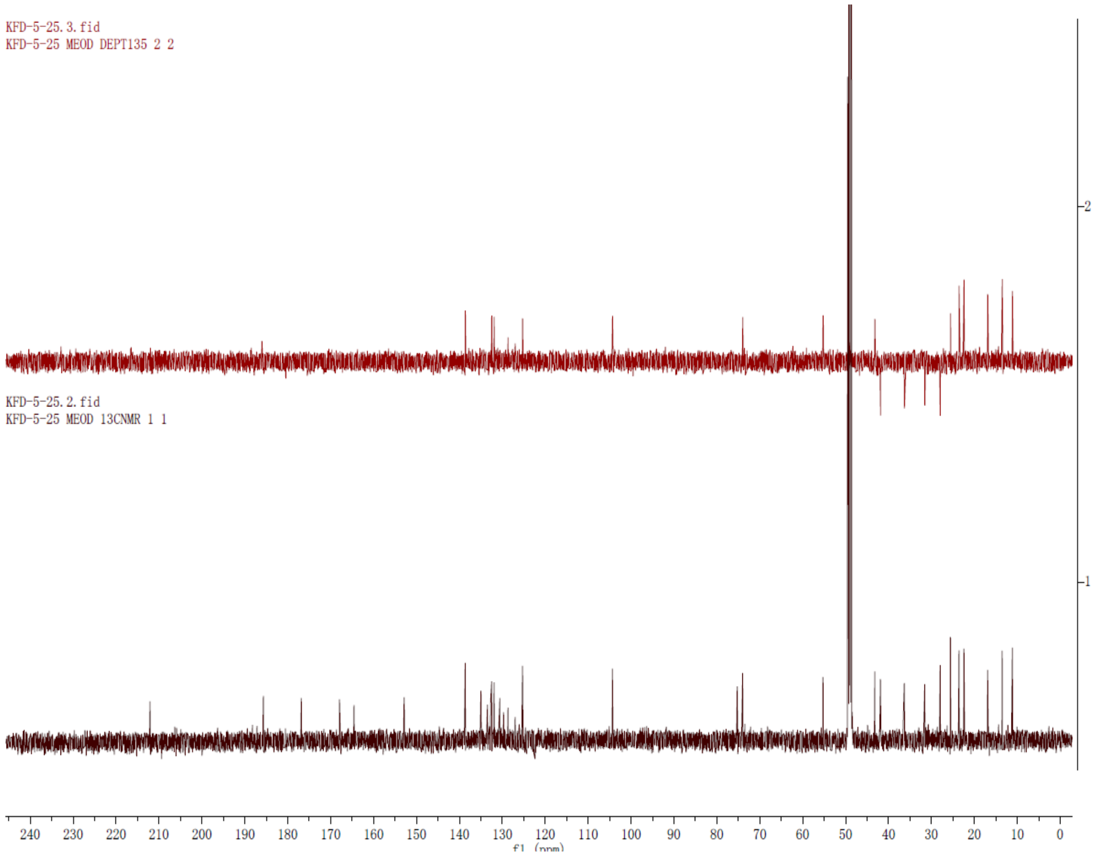

Figure S5-3. The DEPT spectrum of **4**

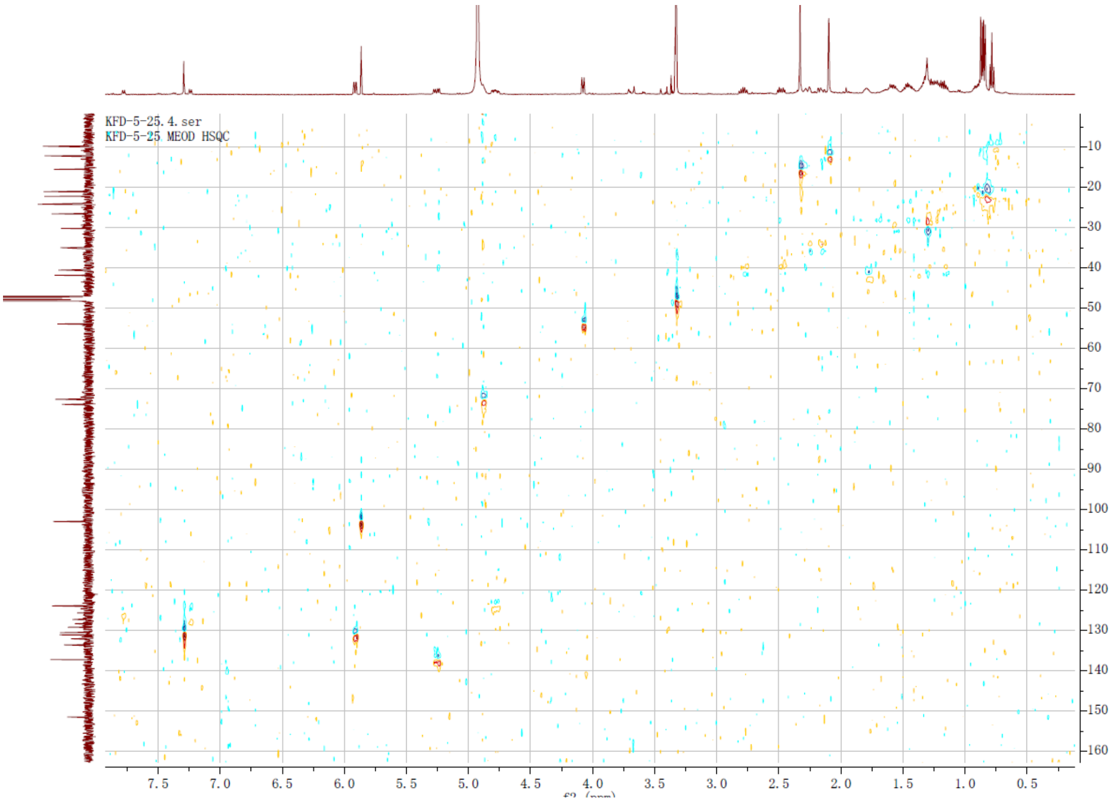

Figure S5-4. The HSQC spectrum of **4**

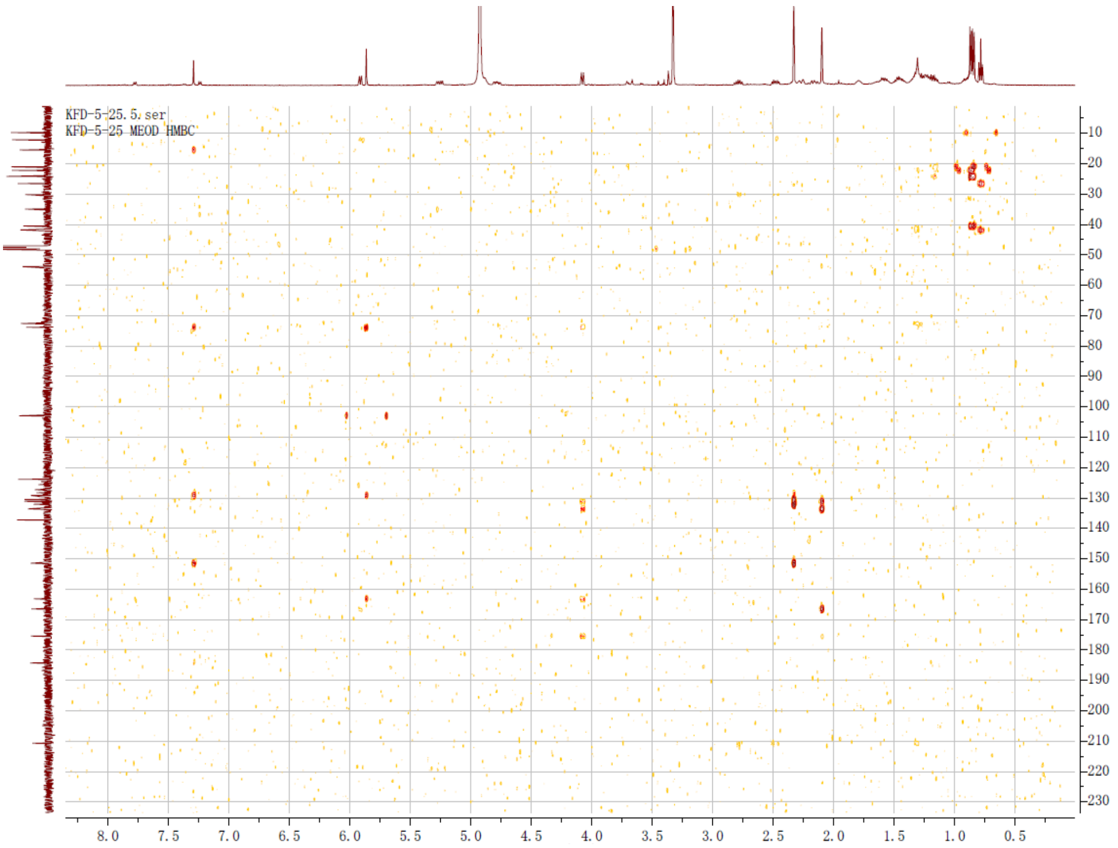

Figure S5-5. The HMBC spectrum of **4**

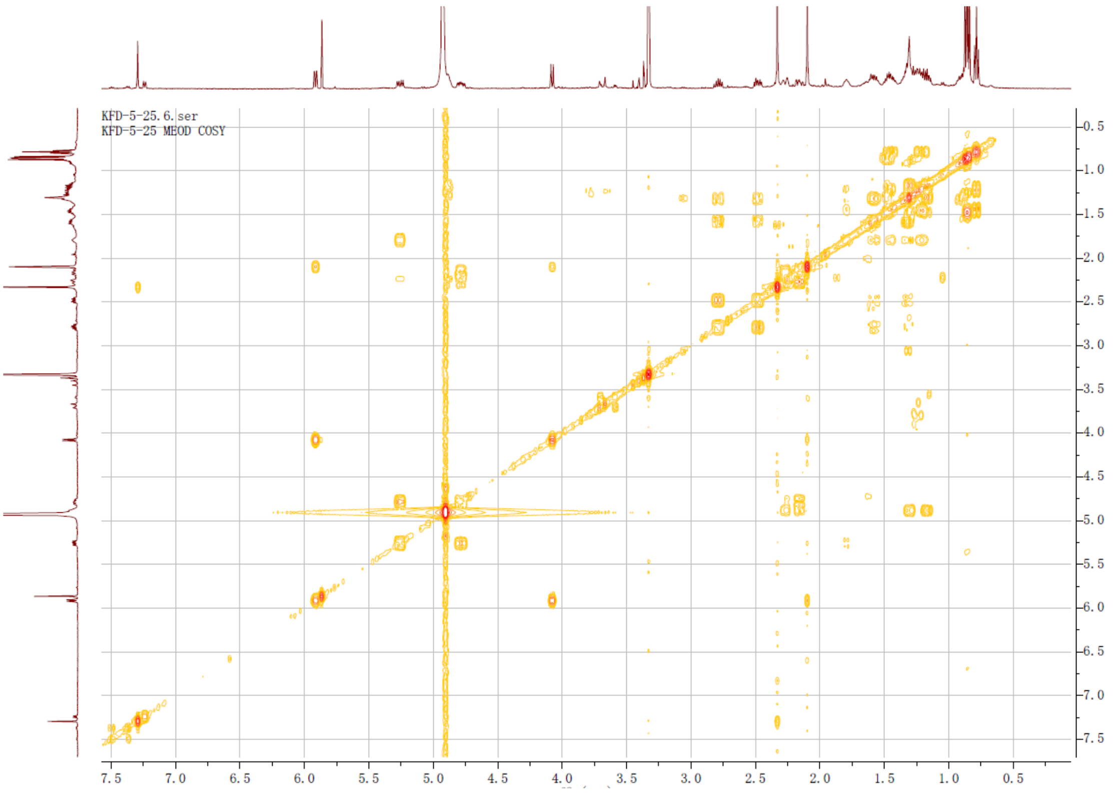

Figure S5-6. The ^1H-^1H COSY spectrum of **4**

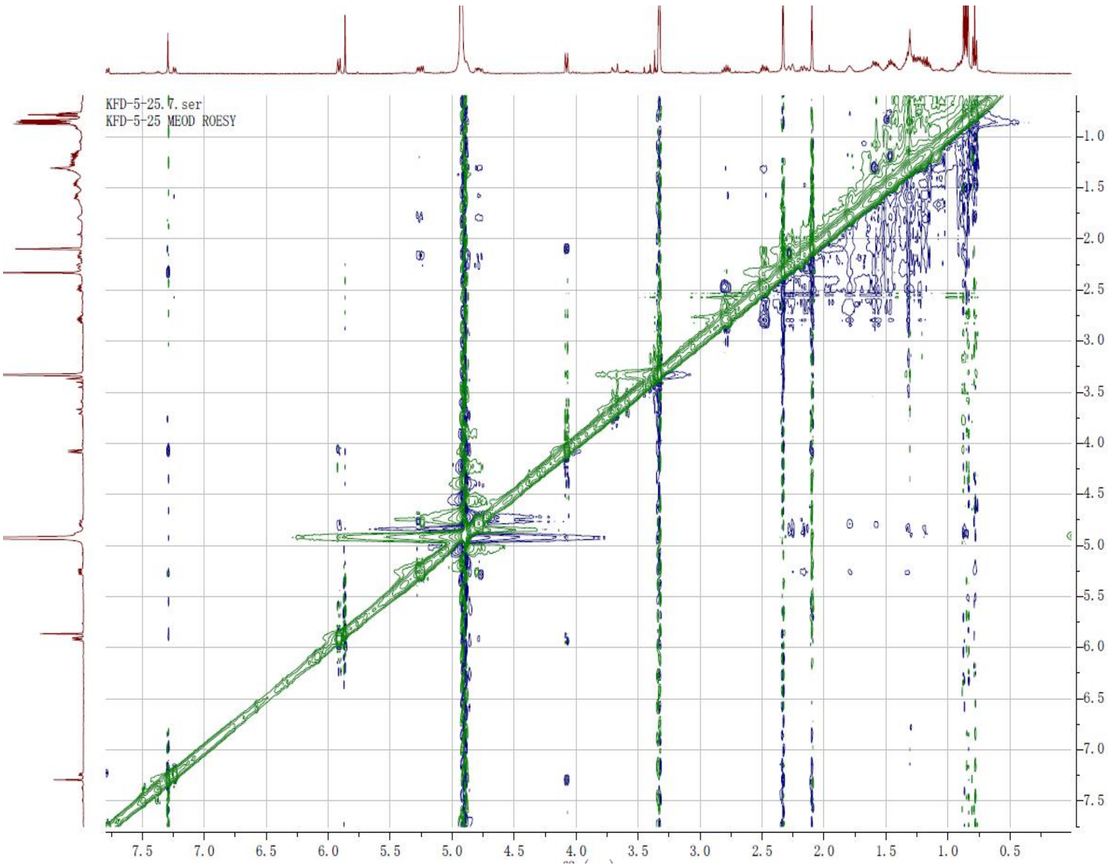

Figure S5-7. The ROESY spectrum of **4**

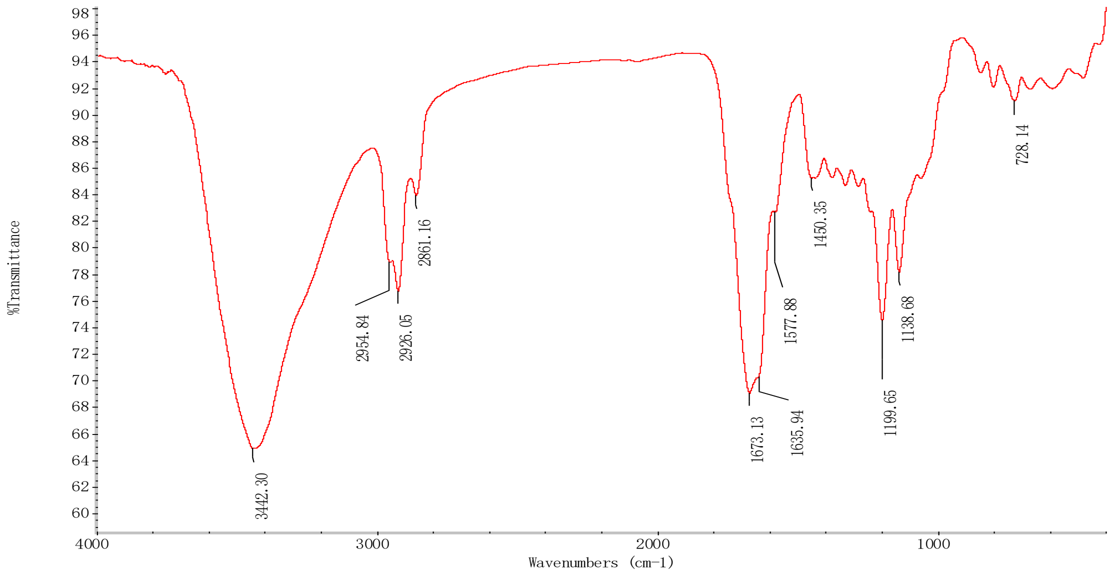

Figure S5-8. The IR spectrum of **4**

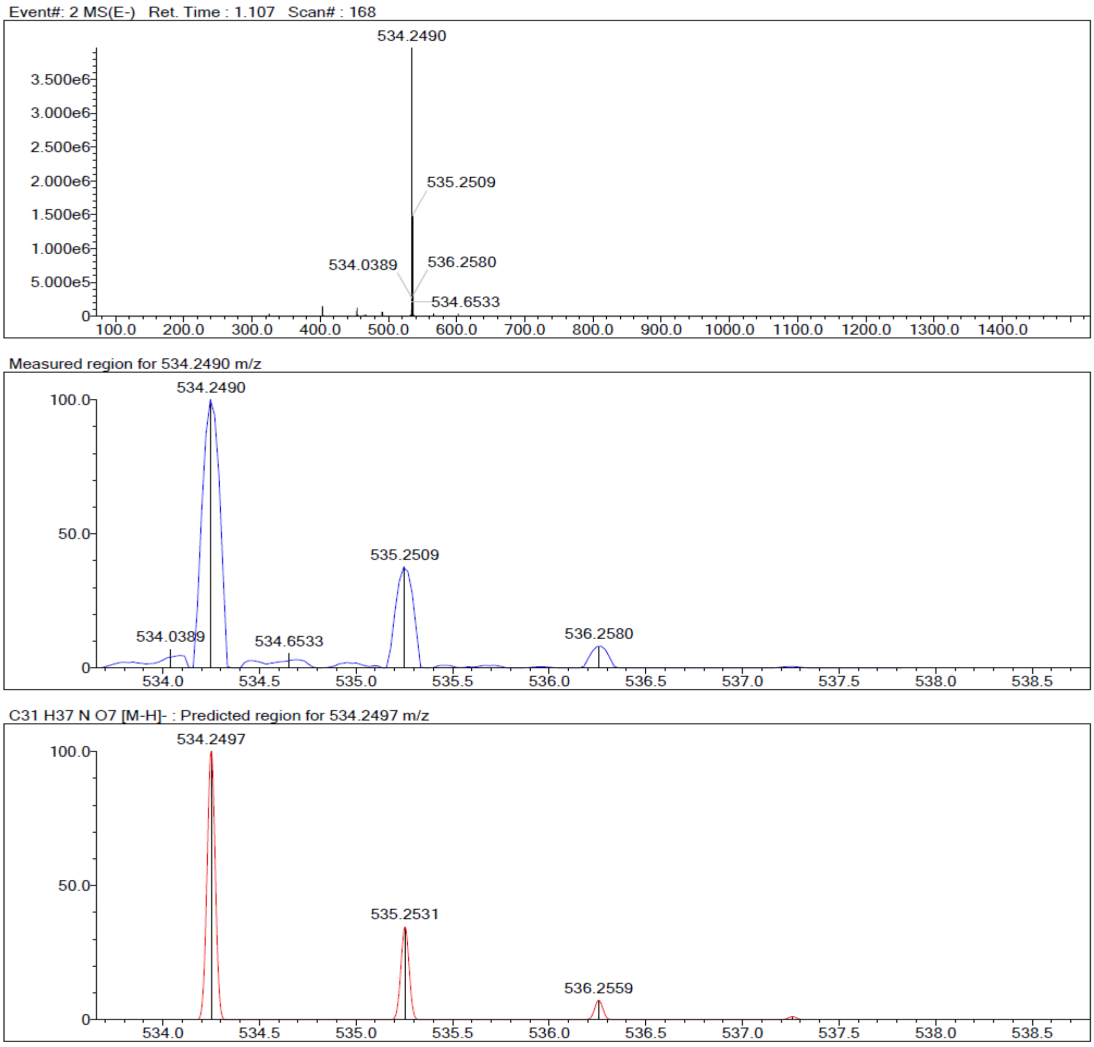

Rank	Score	Formula (M)	Ion	Meas. m/z	Pred. m/z	Df. (mDa)	Df. (ppm)	Iso	DBE
3	87.81	C31 H37 N O7	[M-H]-	534.2490	534.2497	-0.7	-1.31	88.50	14.0

Figure S5-9. The HRESIMS spectrum of **4**
